# Exploring online public survey lifestyle datasets with statistical analysis, machine learning and semantic ontology

**DOI:** 10.1038/s41598-024-74539-6

**Published:** 2024-10-15

**Authors:** Ayan Chatterjee, Michael A. Riegler, Miriam Sinkerud Johnson, Jishnu Das, Nibedita Pahari, Raghavendra Ramachandra, Bikramaditya Ghosh, Arpan Saha, Ram Bajpai

**Affiliations:** 1 Department of Information and Communication Technologies, University of Agder, 4879 Grimstad, Norway; 2 Department of Holistic Systems, Simula Metropolitan Center for Digital Engineering, 0167 Oslo, Norway; 3 Department of Digital Technology, STIFTELSEN NILU, 2007 Kjeller, Norway; 4 Department of Behavioral Sciences, Oslo Metropolitan University, 0176 Oslo, Norway; 5 Department of Information Systems, University of Agder, 4630 Kristiansand, Norway; 6 Department of Software Development, Maulana Abul Kalam Azad University of Technology, Kolkata, 700064 India; 7 Department of Information Security and Communication Technology, Norwegian University of Science and Technology, 2815 Gjøvik, Norway; 8 Department of Finance, Symbiosis Institute of Business Management, Symbiosis International (Deemed University), Bengaluru, 560100 India; 9 Department of Finance, Harper Adams University, Shropshire, TF10 8NB UK; 10 Department of Pharmaceutical Chemistry, Bharat Pharmaceutical Technology, Tripura, 799130 India; 11 School of Medicine, Keele University, Keele, Newcastle, ST5 5BG UK

**Keywords:** Survey, Datasets, COVID-19, Depression, Machine learning, Semantics, LIME, Computational science, Computer science, Information technology, Scientific data

## Abstract

Lifestyle diseases significantly contribute to the global health burden, with lifestyle factors playing a crucial role in the development of depression. The COVID-19 pandemic has intensified many determinants of depression. This study aimed to identify lifestyle and demographic factors associated with depression symptoms among Indians during the pandemic, focusing on a sample from Kolkata, India. An online public survey was conducted, gathering data from 1,834 participants (with 1,767 retained post-cleaning) over three months via social media and email. The survey consisted of 44 questions and was distributed anonymously to ensure privacy. Data were analyzed using statistical methods and machine learning, with principal component analysis (PCA) and analysis of variance (ANOVA) employed for feature selection. K-means clustering divided the pre-processed dataset into five clusters, and a support vector machine (SVM) with a linear kernel achieved 96% accuracy in a multi-class classification problem. The Local Interpretable Model-agnostic Explanations (LIME) algorithm provided local explanations for the SVM model predictions. Additionally, an OWL (web ontology language) ontology facilitated the semantic representation and reasoning of the survey data. The study highlighted a pipeline for collecting, analyzing, and representing data from online public surveys during the pandemic. The identified factors were correlated with depressive symptoms, illustrating the significant influence of lifestyle and demographic variables on mental health. The online survey method proved advantageous for data collection, visualization, and cost-effectiveness while maintaining anonymity and reducing bias. Challenges included reaching the target population, addressing language barriers, ensuring digital literacy, and mitigating dishonest responses and sampling errors. In conclusion, lifestyle and demographic factors significantly impact depression during the COVID-19 pandemic. The study’s methodology offers valuable insights into addressing mental health challenges through scalable online surveys, aiding in the understanding and mitigation of depression risk factors.

## Introduction

### Overview

By June 15, 2020, the global spread of the COVID-19 pandemic had affected over 200 countries, resulting in more than 7 million confirmed cases and precipitating a profound global health crisis, instigating significant shifts in social dynamics^1^. This crisis has imposed formidable challenges on the healthcare infrastructures of diverse nations, disrupting societal norms and functions at large^1^. Furthermore, the imposition of stringent social distancing measures has engendered considerable psychological distress and burden among the general populace^2–4^, exacerbating vulnerabilities among individuals predisposed to mental health disorders^5^. Initial observations indicate that the repercussions of the pandemic, both health-wise and economically, disproportionately impact socioeconomically disadvantaged populations^5^. Notably, marginalized groups such as the homeless face heightened vulnerability to the virus due to challenges in accessing safe shelter^5^. Similarly, individuals lacking access to essential resources like running water, refugees, migrants, and displaced persons are disproportionately affected by the pandemic’s aftermath, encountering barriers to mobility, diminished employment prospects, and increased instances of xenophobic attitudes^5^. During the initial phase of the COVID-19 pandemic, there was a notable rise in the worldwide prevalence of anxiety and depression, as reported in a scientific publication by the World Health Organization (WHO)^6^. This escalation, amounting to a 25% increase, has underscored the pressing need for heightened attention to global mental health concerns. To address these challenges, 90% of surveyed countries have incorporated mental health and psychosocial support measures into their COVID-19 response strategies. Nonetheless, despite these efforts, significant gaps and apprehensions persist^6^. The surge in mental health issues can be attributed to various factors, with the unparalleled stress induced by social isolation and pandemic-related distress being a primary driver. Additional stressors such as feelings of loneliness, apprehension regarding contagion, diminishment of social networks, experiences of suffering, loss, and financial anxieties have been identified as exacerbating factors contributing to the onset of anxiety and depression during the COVID-19 pandemic^6^.

In India, as of 19:19 CET on January 9, 2023, there were a total of 44,680,094 confirmed cases of COVID-19, with 530,721 reported deaths, spanning from January 3, 2020^7^. Concurrently, recent investigations have highlighted a notable surge in the prevalence of depression and anxiety among the Indian populace amidst the ongoing COVID-19 pandemic^8^. Additionally, global assessments indicate a substantial uptick of 27.6% in the prevalence of major depressive disorders throughout 2020 due to the pandemic^9^. Pre-pandemic estimations suggested that approximately 5% of adults worldwide grapple with depression^10^. Notably, depression stands as a significant contributor to the global disease burden and remains a principal cause of disability. Depression, characterized by persistent feelings of sadness and diminished interest, is a psychological disorder that significantly impacts individuals’ emotional and physical well-being^2–5^. Manifesting in alterations in mood, cognition, and behavior, depressive states can detrimentally affect various aspects of daily functioning, including performance at work, school, and in interpersonal relationships^10–13^. This disorder arises from a multifaceted interplay of social, psychological, and biological factors^10^. Individuals who have encountered adverse life events, such as job loss or trauma, are at an increased risk of developing depression^10^. Moreover, depression perpetuates a cycle of heightened stress, impaired functioning, and worsening symptoms, exacerbating the overall burden on affected individuals and their living conditions^10^. This study endeavors to explore the interplay between lifestyle and demographic elements and the manifestation of depressive symptoms among the Indian populace amidst the COVID-19 crisis.

### Motivation

Numerous studies have established a correlation between depression and physical health^10^. Specifically, lifestyle diseases are recognized to play a contributory role in both the onset and persistence of depression, and conversely^10^. The lifestyle disruptions, social isolation, and psychological strain induced by the COVID-19 pandemic are anticipated to exacerbate depressive symptoms within the population. Multiple investigations have underscored a robust link between depression, unhealthy lifestyle practices, and COVID-19 infection^14^. Notably, alterations in lifestyle behaviors, encompassing daily routines, dietary patterns, body composition, and socio-cultural perspectives, have been documented in response to COVID-19 infection. Concurrently, studies have identified prevalent psychosocial distress among affected individuals^15^. Additionally, evidence suggests an association between weight gain, reduced physical activity, and the pandemic’s psychosocial repercussions^15^. The impact of COVID-19 extends beyond sedentary lifestyle tendencies to encompass disruptions in healthy dietary habits^15,16^. In the context of India, the COVID-19 lockdown period has precipitated marked increases in screen time usage alongside reduced consumption of unhealthy foods^17^. Moreover, Indian youth have reported significant disruptions across various facets of life, notably school performance, social engagements, and physical well-being, owing to the pandemic’s effects^15^. With lifestyle-related disorders posing a substantial threat to public health, understanding their correlation with depression becomes imperative. The unprecedented circumstances brought about by the COVID-19 pandemic have heightened the urgency to investigate the intricate relationship between lifestyle choices, demographic factors, and mental health outcomes. By unraveling these connections, interventions and strategies can be devised to mitigate the impact of depression, particularly in vulnerable populations.

In the realm of data science and public health research, the exploration of online public survey lifestyle datasets has garnered significant attention. Understanding the intricacies of lifestyle factors, demographic characteristics, and their correlation with mental health outcomes, particularly amidst the COVID-19 pandemic, is imperative for devising effective intervention strategies. Our review found a very limited number of articles on a similar topic. This sets the stage for reviewing the inputs of various studies in elucidating the complexities of lifestyle datasets and their implications for public health research. The existing literature encompasses a range of studies focusing on different aspects of exploring online public survey lifestyle datasets during COVID-19. Dolton et al.^18^ discuss the challenges of statistical modeling in managing the COVID-19 crisis. They propose a taxonomy of different models and suggest how they can be used together to overcome limitations. Welte et al.^19^ conducted a systematic review to identify clinical trials of pharmacological interventions for COVID-19. They emphasize the importance of tailoring interventions to disease stage and severity for maximum efficacy. Surkalim et al.^20^ provided the pooled prevalence of loneliness for adolescents and adults in different regions. According to them, loneliness is prevalent worldwide, yet data coverage varies significantly, posing equity concerns. Temporal trends in loneliness lack sufficient evidence, hindered by data scarcity and methodological disparities. Addressing these challenges requires integrating loneliness into broader health surveillance and utilizing standardized measurement tools across diverse demographics and regions.

This limited volume of studies collectively contributes to our understanding of online public survey lifestyle datasets, each bringing unique perspectives and insights to the field. The key findings underscore the significance of ensuring data quality and anonymity in online surveys. Moreover, the studies discuss prevalent trends and challenges encountered in handling lifestyle datasets. Furthermore, they provide insights into machine learning approaches utilized for data analysis and highlight the relevance of semantic modeling techniques in survey research. The key limitations of the related work include a lack of discussion on statistical and machine learning techniques, limited focus on machine learning and semantic modeling approaches, absence of coverage on statistical analysis techniques and semantic modeling methods, and a deficiency in discussing statistical and machine learning methods for data analysis. We have addressed such limitations in this paper; therefore, the study focus is new, and the contribution is novel.

### Aim of the study

In the present study, we conducted an online survey during the second wave of the COVID-19 pandemic in India, spanning from June 2021 to August 2021, to gather data pertaining to lifestyle, habits, and social activity from a substantial sample size of participants. Subsequently, we subjected the acquired dataset to rigorous statistical analyses, employing data visualization techniques along with both supervised and unsupervised machine learning algorithms. Through this comprehensive approach, we aimed to discern significant features associated with self-reported depressive health conditions among the respondents. Notably, our focus remained on capturing high-level and generalized indicators of depressive tendencies, rather than clinical symptoms indicative of a diagnosable depressive state.

The analytical endeavors presented in this paper prioritize a data-centric perspective over a clinical one. The primary objective of this study is to identify and analyze potential lifestyle and demographic determinants associated with depressive symptoms among individuals in India during the COVID-19 pandemic. By conducting an extensive online survey in Kolkata, India, and employing advanced statistical and machine learning techniques, this research aims to discern patterns, predictors, and clusters within the dataset. Additionally, the study seeks to semantically represent the acquired knowledge through an ontology model, facilitating a deeper understanding of the complex interrelationships observed. Through this comprehensive approach, the study endeavors to contribute valuable insights into combating depression amidst challenging societal and environmental conditions.

### Research questions

**Fig. 1:**
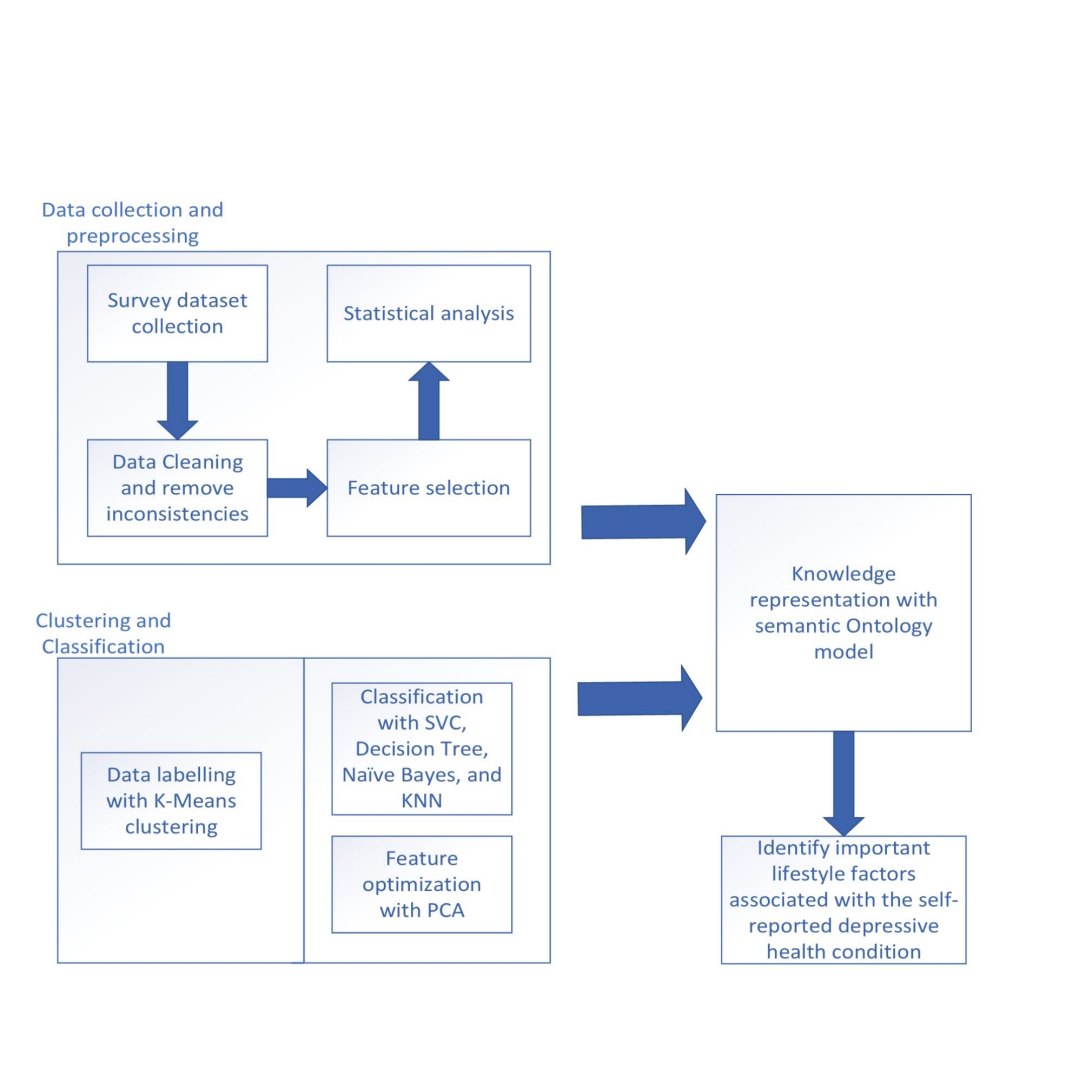
The adopted process in this study.

For data collection, we selected Kolkata, a prominent metropolitan city in India, as our primary study location. Through this focused approach, we aimed to address the following research questions (RQs), the overarching methodology of which is delineated in (Fig. 1).

#### RQ1

*What methodologies can be employed to effectively conduct an online survey for gathering personal*,* behavioral*,* lifestyle*,* habitual*,* and social data from individuals amidst the backdrop of the COVID-19 pandemic*,* and what are the inherent advantages and limitations encountered during the survey process?*

#### RQ2

*What analytical approaches can be utilized to discern significant factors associated with self-reported depressive health conditions within the survey dataset?*

#### RQ3

*What strategies can be employed to proficiently label the dataset in preparation for subsequent analysis?*

#### RQ4

*How can the knowledge extracted from the dataset be semantically represented for enhanced comprehension and interpretation?*

Overall, this study investigates the relationship between lifestyle factors, demographics, and depressive symptoms in public survey data during COVID-19. It begins with a global overview of the pandemic’s impacts, motivating the exploration of these relationships. Using an online survey in Kolkata, diverse data was collected. Rigorous statistical analyses and machine learning techniques aim to identify factors associated with depression. Moreover, an ontology model is employed for semantic representation, contributing to the understanding and mitigation of depression in India amid the pandemic.

## Methods

This study diverges from the conventional framework of a randomized controlled trial (RCT) typically employed in clinical investigations. Instead, it centers on an elucidative analysis and statistical appraisal of anonymized data acquired through random and voluntary participation. Consequently, adherence to the Standards for Reporting Implementation (StaRI) checklist was ensured, as detailed in Supplementary File 1. The collection of data adhered rigorously to pertinent guidelines and regulations, as outlined in the “Ethics Approval and Consent to Participate” Section within the Declarations. Our data collection endeavors primarily aimed at capturing demographic and lifestyle information pertinent to depressive manifestations during the COVID-19 pandemic for subsequent statistical scrutiny. The analytical approach adopted in this study prioritizes the exploration of data-centric insights over clinical delineations. To this end, depression assessments relied on self-reported measures and validated non-clinical scores indicative of depression diagnosis thresholds. In the ensuing analysis, we conducted statistical examinations of the amassed dataset, revealing correlations between certain lifestyle factors and depressive conditions.

Prior to commencing experimentation, all protocols were subjected to scrutiny and approval by the internal ethical board of the Simula Metropolitan Center for Digital Engineering (SimulaMet), followed by endorsement from the Norwegian ethical entity, the Regional Ethical Committee (REK) (accessible at https://rekportalen.no/#home/REK), under reference number #614,685. Stringent adherence to the stipulations of the General Data Protection Regulation (GDPR) was observed throughout the study duration.

### Study characteristics

Internet-based surveys are widely employed for data acquisition, enabling survey questions to reach designated participants via the World Wide Web (WWW)^21–26^. They utilize diverse channels such as email, website integrations, and social media platforms to provide respondents access to online surveys. Table 1 outlines the six primary characteristics that encapsulate our online survey framework.

**Table 1:** The attributes of our online survey.

Attributes	Our approach
Purpose	The purpose of this online survey is to collect personal, behavioral or lifestyle, habitual, and social data from individuals during the COVID-19 pandemic.
Appropriate research design	An online survey from random participants.
Structured questionnaire	A set of 44 multiple-choice questionnaires was prepared with experts and participants.
Defined target sample	Age group > = 18 and < 65; both male and female; digitally literate; understand English; use Internet connectivity; infected or non-infected with COVID-19; willing and motivated.
Collection and analysis of data	Anonymous collection of data. Data analysis with statistical, data visualization methods, and standard machine learning algorithms.
Transparent reporting	Structured representation of knowledge using an ontology.

### Questionnaire preparation for online public survey

Conducting online surveys involves deploying a set of inquiries to a targeted sample via the World Wide Web (WWW). This method is widely adopted for data collection in contemporary research endeavors. Various platforms, such as email, website integrations, and social media channels, facilitate the dissemination of these surveys to respondents. An effective survey design necessitates a judicious combination of open-ended and closed-ended questions to elicit comprehensive responses while maintaining survey efficiency. The formulation of online survey questions is pivotal in eliciting pertinent information from the selected respondents. By incorporating diverse question types, such as multiple-choice, dichotomous, matrix-form, or Likert scale, survey designers can tailor the questionnaire to suit the specific objectives of the study. The alignment between the research objectives and the survey questions is paramount to ensuring the acquisition of valid and meaningful data from online surveys. Moreover, meticulous attention to the structural coherence of survey questions is imperative to capture essential details accurately. A nuanced understanding of the overarching purpose of the online survey facilitates the construction of well-organized and targeted inquiries, thereby enhancing the efficacy and relevance of the data obtained.

The survey questionnaire comprised open-ended multiple-choice inquiries, formulated using Google Forms and disseminated through various social media platforms (e.g., Facebook, LinkedIn, WhatsApp) and electronic mail. Prior to commencing data collection, an online workshop was convened involving e-Health researchers (*n* = 8), e-Health professors (*n* = 5), experts in health policy, survey methodology, and data security (*n* = 4), healthcare professionals (e.g., physicians, nurses) (*n* = 4), a representative sample of participants (*n* = 20), and specialists in computer science and statistics (*n* = 3). This workshop facilitated the development of a comprehensive study roadmap and the formulation of an online questionnaire tailored to survey random participants. Questions within the questionnaire were stratified into three distinct categories based on their method of response: text box-based (for textual input), check box-based (enabling binary selection), and selection box-based (permitting the selection of multiple, non-mutually exclusive elements). Subsequently, collected data was categorized into numeric and categorical groups, with all data uniformly converted into the float64 format to facilitate statistical processing. A detailed description of the survey questions is provided in Supplementary File – 2.

### Data collection

Over a span of three months, data were collected from 1,834 respondents between June 2021 and August 2021. The survey ensured anonymity by omitting any personally identifiable information such as names, emails, or official identifiers like Aadhar ID, Passport Number, Voter ID, PAN ID, or similar government and private identifiers. Participation in the survey was voluntary and contingent upon electronically distributed informed consent, duly signed by the participants. The data collection process involved several steps. Initially, participant data were gathered and stored in Google Drive. Subsequently, the collected data were downloaded in comma-separated values (CSV) format. Following this, all data were transferred to secure storage for subsequent analysis. Data cleaning procedures were then implemented, primarily focusing on outlier identification through box-plot analysis. Entries falling outside the predetermined sample parameters, such as the targeted age range (≥ 18 and < 65), were removed, along with any duplicated entries. Further data refinement included standardizing units across the dataset for consistency.

Body mass index (BMI) calculations were performed based on participants’ height and weight measurements, which were subsequently categorized into four distinct levels of body composition: underweight (0), normal weight (1), overweight (2), and obese (3). Participants were also stratified into three age groups: 0 (age ≥ 18 and age ≤ 40), 1 (age ≥ 41 and age ≤ 64), and 2 (age ≥ 65). To streamline the dataset, redundant features such as height, weight, city, age, and BMI were manually reviewed and removed, limiting the feature count to 40. Detailed information regarding the encoding of categorical variables in the dataset is provided in Supplementary File – 3. The final dataset comprised 1,767 respondents, with 62% being male (standard deviation, σ = ± 21.05, mean, µ = 45.32) and 38% female (σ = ± 20.05, µ = 55.93) following data cleaning and correction processes. The dataset encompassed both numerical and categorical variables, necessitating the encoding of categorical columns into integer labels to facilitate efficient data processing. All survey questions were mandatory, resulting in no missing data instances.

To assess the normality of the dataset, the Shapiro-Wilk test was employed, where a p-value below 0.05 indicated non-normality, leading to the rejection of the null hypothesis. Subsequently, our analysis revealed that the collected survey data did not adhere to a Gaussian distribution. Measures were implemented to uphold dataset security, ensure the anonymity of respondents, and safeguard against unauthorized data manipulation.

### Statistical analysis and data visualization

Correlation analysis was employed to ascertain the presence and strength of relationships between various features within the dataset. The correlation coefficient (r)^27^ served as a metric for quantifying the degree of association between features, ranging from − 1 to + 1. A value of |r| = 1 indicates a perfect linear relationship, where each positive increase in one variable corresponds to a constant proportional increase in the other variable (positive correlation), while |r| = -1 signifies a perfect negative correlation, where an increase in one variable corresponds to a proportional decrease in the other. A value of |r| = 0 denotes no linear relationship between the variables. Given the non-normal distribution of the dataset, Spearman’s rank correlation coefficient was utilized to calculate |r|, enabling the identification of strong correlations with |r| ≥ 0.85 for subsequent elimination of redundant features. The Spearman’s rank correlation coefficient can be calculated as^28^:

1 $$\:{r}_{s}=1-\:\frac{6\sum\:{d}_{i}^{2}}{n({n}^{2}-1)}$$

where r_s_ = Spearman’s rank correlation coefficient, d_i_ = difference between the two ranks of each observation, and n = number of observations.

To find the dependency between the dependent variable and independent variables (or features), we used ANOVA (Analysis of Variance) statistical testing method with the following hypothesis^29^:

- if *p* < 0.05, this means that the categorical variable has significant influence on the numerical variable, and
- if *p* > 0.05, this means that the categorical variable has no significant influence on the numerical variable.

In our analysis, we utilized Python data visualization libraries, including Matplotlib and Seaborn, to visually represent the dataset. Outlier analysis was conducted using box and whisker plots to assess locality, spread, and skewness. Additionally, various plotting methods such as histograms, bar charts, and density plots were used to examine the distribution of the data. Moreover, bar plots with cross-tabulations were employed to visualize dependencies between two features.

### Feature ranking and data labeling with clustering method

Three standard methods were employed in our analysis: SelectKBest utilizing the Chi-Square (χ2) Statistic (chi2), principal component analysis (PCA), and ExtraTreeClassifier. These methods were utilized to assess the fitness score of the features, aiding in their ranking. As our dataset was unlabeled, we utilized the standard K-Means clustering algorithm to label the dataset due to its ease of implementation and rapid convergence. Clustering serves as a widely employed unsupervised learning technique aimed at uncovering hidden patterns or relationships between data points based on shared attributes. It is particularly valuable for drawing insights from large datasets. K-Means clustering was selected for its simplicity and efficiency, especially in scenarios involving large variable sets, as it converges more quickly than hierarchical clustering and yields tighter clusters. The optimal “K” value was determined through Silhouette scoring and the Elbow method. Notably, K-Means clustering is sensitive to scaling and follows the expectation-maximization method for problem resolution. In this method, data points are assigned to clusters to minimize the sum of squared distances between the data points and centroids. The resultant clustering introduced an additional predictor column, yielding a total of 41 features (40 independent and one dependent). The optimum number of clusters finding problem in K-Means clustering can be defined as^30–32^:

2 $$\:\text{arg}S\text{min}\sum\:_{i=1}^{k}\sum\:_{x\in\:{S}_{i}}\left|\left|x-\:{\mu\:}_{i}\right|\right|$$

Where S = set of observations, k = number of sets of predictors, x = observation data points, and $\:{\mu\:}_{i}$ = mean of points in $\:{S}_{i}$.

The pseudo code for finding the best “K” value with data label is stated as follows:

**Table:** 

Pseudo code “K”-value determination based on clustering for data labeling
*Step-1: Define input parameters - data*,* max_clusters = 10*,* scaling*$\:\in\:$*{True*,* False}*,* visualization*$\:\in\:$*{True*,* False}*,* and metric=’euclidean’* *Step-2: Define list - n_clusters_list*,* silhouette_list* *Step-3: if (scaling = = True) Then* *scalar = convert_to_min_max (data)* *else* *scalar = data* *Step-4: For n_c = 2 to max_clusters + 1 do* *kmeans_model = KMeans(n_clusters = n_c).fit(scalar)* *labels = find_labels(kmeans_model)* *n_clusters_list.append(n_c)* *silhouette_list.append(silhouette_score(scalar*,* labels*,* metric = metric))* *End* *Step-5: Cross-verification of “K” value with the Elbow method.* *Step-6: Find the best parameters based on defined lists* *Step-7: Perform data labeling with the best model* *Step-8: Visualize the best Clustering corresponds to Number of clusters (n_c) and Silhouette score.*

### Data classification

In this study, a pipeline methodology was employed, integrating Principal Component Analysis (PCA) with conventional machine learning algorithms for classification purposes. PCA aims to reduce the dimensionality of datasets that have numerous correlated variables while striving to retain maximal variance within the dataset. Within this pipeline framework, PCA played a crucial role in identifying the optimal feature set to achieve superior mean classification accuracy, F1-score, precision, recall, and Matthews Correlation Coefficient (MCC) value. The calculation of performance metrics is outlined in accordance with prior literature^33,34^:

3 $${\text{Accuracy }}\left( {\text{A}} \right){\text{ }} = \frac{{\left( {{\text{TP}} + {\text{TN}}} \right){\text{~}}}}{{\left( {{\text{TP}} + {\text{FP}} + {\text{FN}} + {\text{TN}}} \right)}},0 \le \frac{{\left( {\text{A}} \right){\text{~}}}}{{\left( {100} \right)}} \le {\text{1}}$$

4 $${\text{Precision }}\left( {\text{P}} \right) = \frac{{\left( {{\text{TP}}} \right){\text{~}}}}{{\left( {{\text{TP}} + {\text{FP}}} \right)}}$$

5 $${\text{Recall }}\left( {\text{R}} \right){\text{ or Sensitivity }}\left( {\text{S}} \right){\text{ or True positive rate}} = \frac{{\left( {{\text{TP}}} \right){\text{~}}}}{{\left( {{\text{TP}} + {\text{FN}}} \right)}}$$

6 $${\text{Specificity }}\left( {\text{S}} \right) = \left( {{\text{1}} - {\text{Sensitivity}}} \right) = \frac{{\left( {{\text{TN}}} \right){\text{~}}}}{{\left( {{\text{TN}} + {\text{FP}}} \right)}}$$

7 $${\text{F1 score }}\left( {{\text{F1}}} \right) = \frac{{\left( {2{\text{*P*R}}} \right){\text{~}}}}{{\left( {{\text{P}} + {\text{R}}} \right)}},0 \le \frac{{\left( {{\text{F}}1} \right){\text{~}}}}{{\left( {100} \right)}} \le {\text{1}}$$

8 $${\text{Matthew}}^{\prime } {\text{s correlation coefficient }}\left( {{\text{MCC}}} \right) = \frac{{\left( {{\text{TP}}\left( {{\text{TP*TN~}}{-}{\text{~FP*FN}}} \right){\text{~}}} \right){\text{~}}}}{{\surd \left( {\left( {{\text{TP}} + {\text{FP}}} \right)\left( {{\text{TP}} + {\text{FN}}} \right)\left( {{\text{TN}} + {\text{FP}}} \right)\left( {{\text{TN}} + {\text{FN}}} \right)} \right){\text{~}}}}, - {\text{1}} \le \frac{{\left( {{\text{MCC}}} \right){\text{~}}}}{{\left( {100} \right)}}~ \le + {\text{1}}{\text{.}}$$

Where TP: True Positive, TN: True Negative, FP: False Positive, and FN: False Negative.

The following standard machine learning algorithms were used for classification with 5-fold cross validation as they represent most common families of algorithms and work well with limited dataset and multiple features:

- Support Vector Classifier (SVC): A support vector machine^35^ is a supervised linear machine learning algorithm most used to solve classification problems, also known as support vector classification. The SVC algorithm helps to find the best line or decision boundary; this best boundary or region is called a hyperplane. The SVC algorithm finds the closest line point from two classes. These points are called support vectors. The distance between the vector and the hyperplane is called the margin. The goal of SVC is to maximize this margin. The hyperplane with the largest margin is called the optimal hyperplane. A kernel function in SVC is a method for processing data that takes data as input and transforms it into a desired form. It returns the inner product between two points in the standard feature dimension. Usually, the training dataset is transformed so that the non-linear decision surface can be transformed into a linear equation in a higher dimensional space. SVC uses the following kernels – Linear, Gaussian, Gaussian Kernel Radial Basis Function (RBF), Sigmoid, and Polynomial. The mathematical model behind the SVC can be defined as:

$$\:maximize\:f\left(c1,c2,\dots\:.,cn\right)=\:\sum\:_{i=1}^{n}{C}_{i}-0.5\:\sum\:_{i=1}^{n}\sum\:_{j=1}^{n}{y}_{i}{C}_{i}\:\left({x}_{i}\:.\:\:{x}_{j}\right){\:y}_{i}{C}_{j}$$

subject to

9 $$\mathop \sum \limits_{{i = 1}}^{n} C_{i} y_{i} = 0,\;{\text{and}}\;0~\, \le ~\,C_{i} \,~ \le ~\,\frac{1}{{2n\partial }}\forall \,{\text{i}}.$$

b) DecisionTreeClassifier: A decision tree^36^ is a supervised machine learning algorithm that uses a set of rules, like how humans make decisions. Decision trees capture knowledge in the form of a tree, which can also be rewritten as a discrete set of rules for better understanding. The intuition behind decision trees is that dataset creates yes/no questions and keeps partitioning the dataset until we isolate all data points belonging to each category. Both the Gini coefficient and entropy are measures of node impurity for classification. The Gini index has a maximum impurity of 0.5 and a maximum purity of 0, while entropy has a maximum impurity of 1 and a maximum purity of 0. Multi-class nodes are impure, while single-class nodes are pure. Entropy is more expensive to compute because of the logarithm in the equation.
c) Naïve Bayes: The naive Bayes^37^ algorithm is a supervised learning algorithm based on the Bayes theorem for solving classification problems. Naive Bayesian classifiers are one of the simplest and most effective classification algorithms that help build fast machine-learning models that can make quick predictions. It is a probabilistic classifier, which means it makes predictions based on the probability of the object. It assumes that a particular trait occurs independently of other characteristics. The mathematical expression of Naïve Bayes is:

10 $$\:P\:\left(A\right|B)\:=\:\frac{P\left(B\right|A\left)P\right(A)}{P\left(B\right)}\:\:\:and\:P(B)\:=\:\sum\:_{y}P\left(B\right|A\left)P\right(A)$$

Where, $\:P\:\left(A\right|B)$ = Posterior, $\:P\left(B\right|A)$ = Likelihood, $\:P\left(A\right)$ = Prior, $\:P\left(B\right)$ = Normalizing constant

d) K-Nearest Neighbor (KNN): K-Nearest Neighbors^38^ is one of the simplest machine-learning algorithms based on supervised learning techniques. It stores all available data and classifies new data points based on similarity. It assumes the similarity between new cases/data and available cases and places new cases in the category most like general categories. It means that when new data comes in, it can be easily classified into the appropriate type using the KNN algorithm. It is a nonparametric algorithm, which means it does not make any assumptions about the underlying data. The lazy learner algorithm is called because it does not immediately learn from the training set but instead stores the dataset and performs operations on the dataset while classifying. The mathematics behind KNN is:

11 $$P(y = j|X = x)~ = ~\frac{1}{K}~*~\mathop \sum \limits_{{i~ \in ~A~}} I\left( {Y^{i} ~ = ~j} \right)$$

We split the dataset with a train, validation, and test ratio of 60:20:20 (and a random state of 42). We used the grid-search technique to find the best parameters for the classifier. Finally, we used the validation and learning curve for model verification.

### Classification explanation

In this study, we used the LIME algorithm^39^ to generate explanations that are easily understandable to humans, enabling users to gain insights into the model’s decision-making process. These explanations aim to help understand why a model made a particular prediction for a specific instance or observation. LIME works by approximating the behavior of a black-box model in the local vicinity of the instance being explained.

LIME offers instance-level explanations, focusing on individual predictions rather than overall model behavior. It is versatile across various machine learning models, including deep neural networks and ensemble methods, ensuring interpretability regardless of model complexity. LIME generates saliency maps using a gradient-based approach, where brighter colors signify higher feature influence. By training interpretable models on perturbed data, LIME approximates black-box model behavior locally. It visualizes feature importance using color codes, with brighter colors denoting greater influence. This approach enhances model interpretability, which is crucial for understanding complex predictions. Here’s a description of LIME color codes used in this context –

- Orange color: In the feature importance visualization, orange is commonly used to highlight features that have a significant impact on the model’s prediction for the instance being explained. These features are considered the most influential in determining the output of the black box model.
- Red color: Red may indicate significant importance but slightly less than orange. Features highlighted in red still hold considerable influence on the prediction for a specific class but may not be as dominant as those depicted in orange.
- Blue color: Conversely, blue is used to denote features that have a minimal or negligible effect on the model’s prediction. These features are deemed less important in influencing the output of the black-box model and may have little impact on the final prediction.
- Green color: LIME uses green to symbolize the original prediction made by the black-box model for the instance under consideration. This serves as the baseline for comparison with the explanations provided by LIME.
- Yellow color: In saliency maps, yellow is commonly used to denote regions of high saliency, indicating areas of the input space that have the greatest influence on the model’s prediction. These regions are considered crucial in shaping the model’s decision for the instance under consideration.
- Purple color: Purple is used to denote regions of low saliency, indicating areas of the input space that have minimal influence on the model’s prediction. These regions are considered less important in determining the output of the black-box model and may have little impact on the final prediction.

### Ontology modeling

We developed an ontology model using the Protégé (v. 5.x) open-source software to encapsulate insights derived from the survey dataset. Visualization of the ontology was achieved through the OWLViz tool within Protégé. In this object-oriented representation, owl.

serves as the overarching parent class, with arrows denoting hierarchical relationships (IS-A) between the concepts^40,41^. The ontology encompasses various elements, including classes, objects, properties, relationships, and axioms. Properties are categorized into two types: ObjectProperties and DataProperties, each with defined domain scopes, restriction rules, filters, and types such as Some (existential), Only (Universal), Min (minimum cardinality), Exact (exact cardinality), and Max (maximum cardinality). A detailed explanation of our designed ontology is provided in (Textbox 1). Our OWL ontology adheres to distinct knowledge representation phases, including abstraction for rule mapping, abduction for hypothesis generation, deduction for operator-reductor rules, and induction for generalization. The object-oriented class structure of the OWL ontology is presented in Supplementary File 4, formatted in “TTL” for improved readability. The following is a general step for semantic Ontology design and development used in this study:

- Domain identification to define the scope of the Ontology and model associated concepts (classes) and their associations.
- Knowledge and requirement gathering on the domain, including relevant literature, expert opinions, and existing ontologies. This information is used to identify concepts and relationships that need to be modeled in ontology.
- Defining of Ontology structure with classes, properties (object and data), axioms, and relationships that will be used to represent the concepts and their interrelationships.
- Ontology development with well-established editors or software tools. This involves creating the classes, attributes and relationships defined in the previous step and adding instances to the ontology to illustrate the concepts.
- Structural consistency checking of the Ontology.
- Validation of the Ontology with real or simulated dataset for individuals (objects) to ensure that it accurately represents the domain and can be used for its intended purpose. This may involve using ontologies to perform tasks such as classification, retrieval, or inference.
- Refine and update the Ontology with new knowledge. It is important to maintain the Ontology and ensure it remains current and useful.

**Textbox 1:** The ontology expression.

An ontology can be defined as a tuple Ω = {X, R}, where X is the set of concepts or classes, and R is a set of relations. L = Levels (О_h_) = Total number of levels in the ontology hierarchy, 0 ≤ n ≤ L, where n⋲ Z^+^ and *n* = 0 represents the root node. X_n, j_ = a model classifying О at a level n; where, j ⋲ {0, 1, …., |X_n_|} |X| = Number of instances classified as class X E = Edge (X_n, j_, X_*n*−1, k_) = edge between node X_n, j_ and its parent node X_*n*−1, k_ We have used the concept and represented our ontology with four tuples: O = {X_a_, R, I, A} X_a_: {X_a1_, X_a2_, …., X_an_} represents “n” concepts or classes and each X_a.i._ has a set of “j” attributes or properties A_i_ = {a_1_, a_2_,……., a_j_} provided n, i, j ⋲ Z^+^. R: A set of binary relations between the elements of X_a_. It holds two subsets – a. H: Inheritance relationship among concepts b. S: Semantic relationship between concepts with a domain and range I: Represents a knowledge base with set of object instances. A: Represents a set of axioms to model O. A includes domain specific constraints to model an Ontology with X_a_, R, and I.	

## Results

### Experimental setup

Python 3.9.15 libraries, including pandas (v. 1.5.2), NumPy (v. 1.22.4), SciPy (v. 1.7.3), Matplotlib (v. 3.6.2), Seaborn (v. 0.12.0), Plotly (v. 5.11.0), and scikit-learn (v. 1.1.3), were used for data preprocessing and machine learning model development. The Python environment was set up on the Windows 10 operating system using the Anaconda distribution, with Jupyter Notebook (v. 6.5.2) serving as the platform for development, model analysis, and data visualization. The computational setup included 16GB RAM and a 64-bit architecture. Due to the dataset’s modest size, computational tasks were performed on a CPU.

### Analysis

#### Respondent type

The ‘Google Online Survey Form’ was distributed among approximately 3,200 interested participants, resulting in responses from 1,834 individuals (response rate: 57.31%). Subsequently, data meeting predefined inclusion and exclusion criteria were retained for 1,767 respondents (96.34%). Among these, 671 (38%) identified as female, while 1,096 (62%) identified as male. Notably, 698 respondents (39.5%) reported experiencing depressive symptoms during the measurement period, with the remaining 1,067 respondents (60.5%) reporting no such symptoms. Analysis of respondent demographics revealed a mean age of xx. Specifically, 724 respondents were aged ≥ 18 and ≤ 40, 534 were aged ≥ 41 and ≤ 65, and 509 were aged over 65. Regarding body composition, 208 respondents were classified as underweight, 559 as normal weight, 338 as overweight, and 662 as obese. Eighteen respondents acknowledged developing addiction during the COVID-19 pandemic. Additionally, 277 respondents exhibited inclinations towards unhealthy lifestyles, while 585 were identified as adhering to a sedentary lifestyle during the pandemic.

Socioeconomic characteristics indicated that most participants belonged to the middle class (approximately 52%), followed by the upper middle class (approximately 15%). Respondents with a bachelor’s degree constituted the largest educational subgroup (approximately 47%), followed by those with a master’s degree (approximately 25%). Employment status varied, with approximately 32% of participants engaged in full-time employment, while approximately 17% identified as unemployed and/or homemakers. Furthermore, approximately 83% of respondents reported possessing medical insurance, with approximately 55% engaging in regular physical activities. The prevalence of negative lifestyle factors over the past five years was reported by approximately 83.6% of participants. Among health issues, high blood pressure, sleeping disorders, overweight and obesity, depression, cardiovascular diseases, high cholesterol levels, and type II diabetes exhibited descending prevalence within the participant cohort. On average, respondents required 15–18 min to complete the survey questionnaire.

#### Feature analysis

Based on the p-value obtained from the Shapiro test, it is evident that our features do not follow a Gaussian distribution. Subsequently, correlation analysis indicated a modest level of correlation among the features. The relationship between the features and their impact on the depressive state was examined through ANOVA testing, and the results are presented in Table 2. For this analysis, we utilized the linear Ordinary Least Squares (OLS) Model and employed the “anova_lm” function for ANOVA assessment. Notably, we used a Type 2 ANOVA data frame for this purpose. Features exhibiting a significance level of “p < 0.05” were identified as exerting influence on the depressive health state. Through ANOVA testing, differences between groups were discerned, thereby highlighting key features within the dataset.

**Table 2:** The ANOVA testing results of features on the depressive state feature.

Features	sum_sq	Degree of freedom (df)	F	PR(> F)	*p* < 0.05
Age_group	1.16	2.0	2.43	0.088265	False
Gender	4.03	1.0	16.99	0.000039	True
Dependant	1.78	1.0	7.46	0.006357	False
Is_Employed	1.45	1.0	6.09	0.013683	True
Is_Medically_Insured	4.06	1.0	17.14	0.000036	True
Visit_Gym	0.6	1.0	2.55	0.110682	True
Week_Days_sleep_time_avg	2.29	1.0	9.61	0.001956	True
Body_Composition	2.00	1.0	8.43	0.003717	True
Educational_Level	1.60	1.0	6.71	0.009626	False
Economic_Status	0.015	1.0	0.06	0.800787	False
Duration_of_daily_exercise	5.10	1.0	21.59	0.000004	True
Duration_of_daily_walking	5.52	1.0	23.4	0.000001	True
Smoking_Habit	0.18	1.0	0.78	0.3763	False
Habit_of_Snus	0.04	1.0	0.02	0.895631	False
Habit_of_Alcohol	0.2	1.0	0.84	0.360532	False
Habit_of_Energy_Drinks	1.42	1.0	5.97	0.014653	True
Habit_of_Paan_Masala	2.19	1.0	9.23	0.002407	True
Mobile_Application_for_Activity_Tracking	0.67	1.0	2.80	0.093994	False
Mobile_Application_for_Diet_Tracking	0.98	1.0	4.10	0.04296	True
Social_Participation_Type	0.54	1.0	2.27	0.131359	False
Social_Participation_Duration	0.00005	1.0	0.0002	0.988741	False
Consumption_of_Vegetables	2.4	1.0	10.12	0.001492	True
Consumption_of_Fruits	2.4	1.0	10.03	0.001563	True
Consumption_of_Junk_Fried_Foods	2.6	1.0	10.3	0.000964	True
Consumption_of_Sweets	0.21	1.0	0.88	0.347617	False
Consumption_of_Red_Meat	0.03	1.0	0.13	0.718481	False
Consumption_of_BBQ_Foods	0.20	1.0	0.84	0.35713	False
Consumption_of_Discritionary_Foods	0.32	1.0	1.33	0.248305	False
Existing_Health_Problems	0.77	1.0	3.26	0.071111	False
Hospitalization_History	0.84	1.0	3.55	0.059593	False
Regular_Physician_Consultation	0.04	1.0	0.17	0.684259	False
Negative_Lifestyle	2.55	1.0	10.7	0.001073	True
Skipping_Diet	4.04	1.0	17.04	0.000038	True
Food_Type	0.58	1.0	2.41	0.120297	False
WFH_for_COVID	2.26	1.0	9.5	0.002054	True
Sedentary_Lifestyle_due_to_COVID	0.25	1.0	1.05	0.305577	False
Unhealthy_Lifestyle_due_to_COVID	0.08	1.0	0.35	0.554336	False
Addiction_due_to_COVID	4.44	1.0	18.73	0.000016	True

### Clustering

The K-Means clustering algorithm iteratively calculates centroids until an optimal configuration is achieved, with the parameter ‘K’ representing the total number of clusters. Common methods used to determine the appropriate ‘K’ value include the Silhouette score and the Elbow method. The Silhouette score assesses the cohesion of an object within its cluster and its separation from neighboring clusters, with values ranging from − 1 to + 1. Higher Silhouette scores indicate better intra-cluster similarity and inter-cluster dissimilarity. Meanwhile, the Elbow method is a heuristic employed to identify the optimal number of clusters by plotting the explained variation against the number of clusters and selecting the ‘elbow’ point on the curve. To facilitate effective clustering, data scaling techniques such as MinMaxScaler are often utilized, which normalize features to a specified range (e.g., [0, 1] or [-1, 1]).

**Fig. 2:**
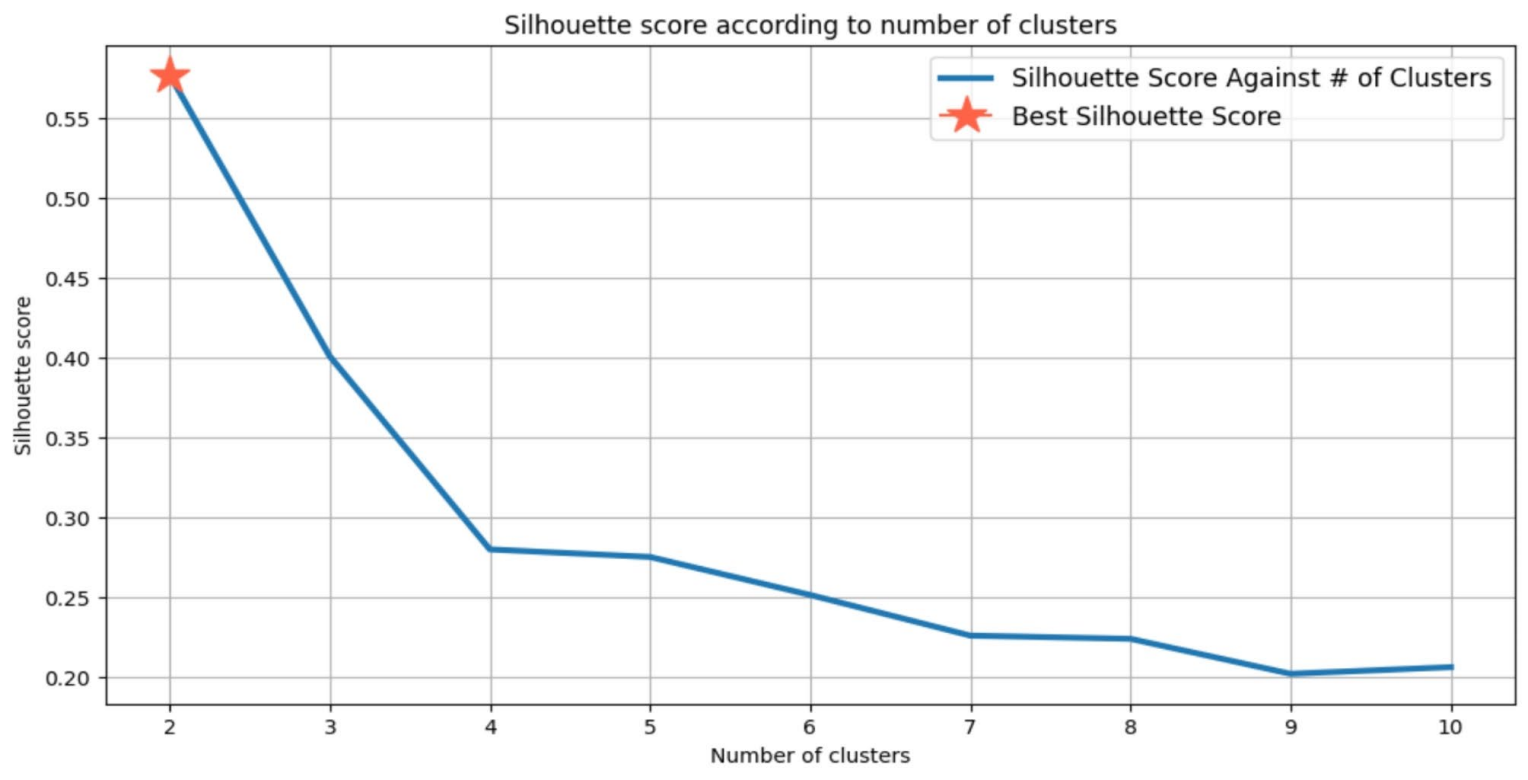
“K” value against Silhouette score without data scaling.

**Fig. 3:**
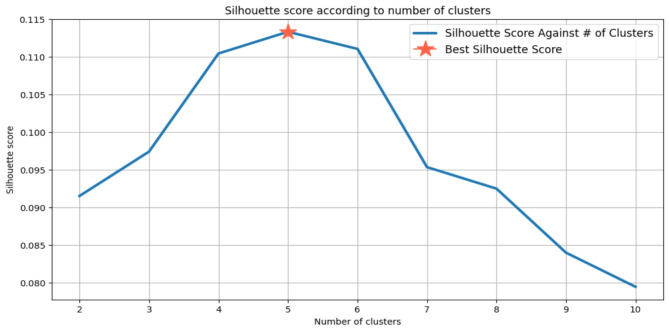
“K” value against Silhouette score with data scaling.

**Fig. 4:**
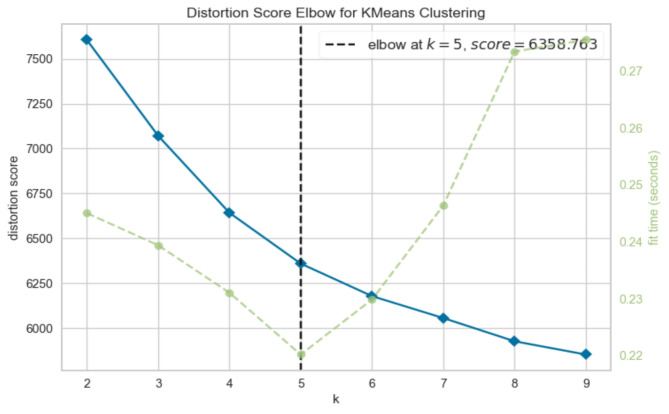
“K” value determination with the Elbow method on scaled data.

In our analysis, we observed that without scaling, the optimal ‘K’ value was determined to be 2, yielding a Silhouette score of 0.58 (see Fig. 2). Conversely, with MinMax scaling applied, the optimal ‘K’ value increased to 5, accompanied by a Silhouette score of 0.11 (see Fig. 3). Cross-verification using the Elbow method on both scaled and unscaled datasets yielded ‘K’ values of 5 and 6, respectively. Based on the convergence of results from the Silhouette score and the Elbow method, we selected a ‘K’ value of 5 for the scaled dataset, as depicted in Fig. 4. The utilization of scaling facilitated the alignment of features on a common scale, enhancing clustering performance. Consequently, the resulting clustering yielded five distinct prediction classes, denoted as {0, 1, 2, 3, 4}. The distribution of instances across these classes was as follows: Class 0 (*n* = 163), Class 1 (*n* = 495), Class 2 (*n* = 255), Class 3 (*n* = 341), and Class 4 (*n* = 513).

### Classification

**Fig. 5:**
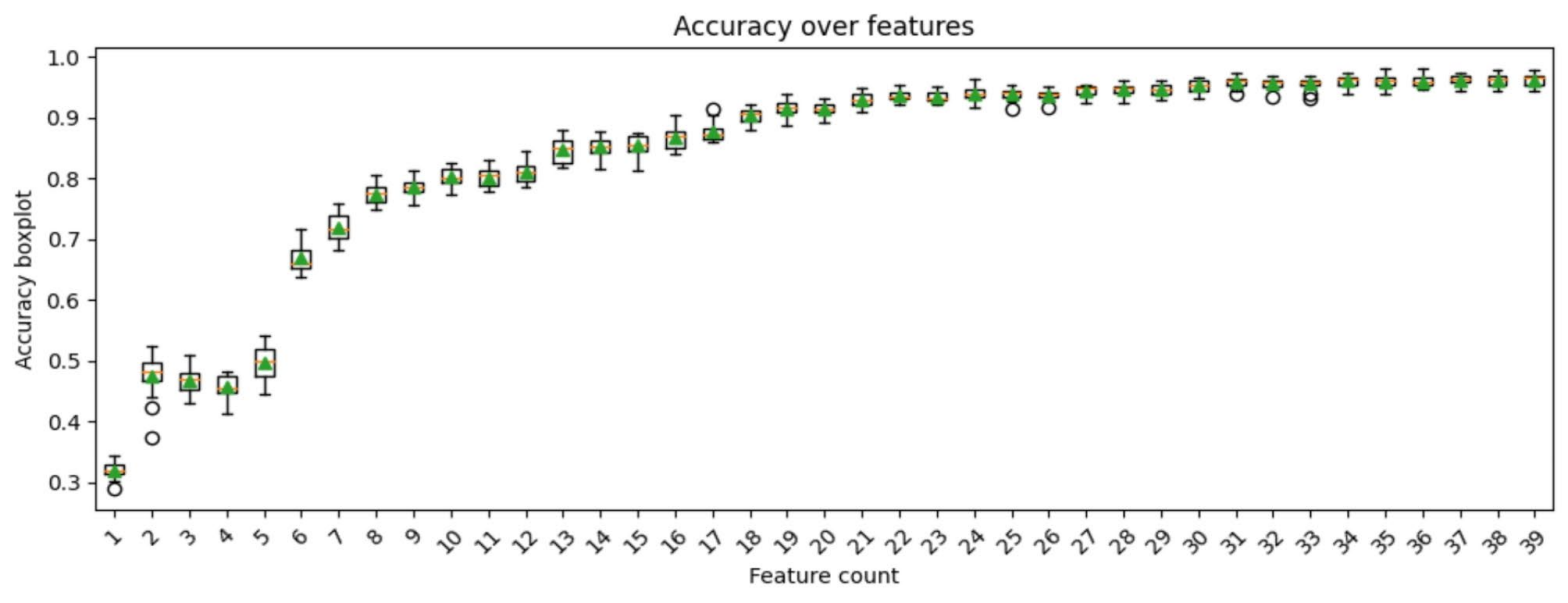
The accuracy score of PCA + SVC pipeline with optimal feature count.

The Principal Component Analysis (PCA) combined with Support Vector Classification (SVC), utilizing a learning rate (C) of 0.01 and gamma (γ) value set to 0.01, yielded the highest accuracy of 96%. This model demonstrated robust performance metrics, including an F1-score of 96%, precision of 95%, recall of 96%, and a Matthews Correlation Coefficient (MCC) of 94%. The pipeline utilized 31 features, with a standard deviation of the accuracy score calculated as 0.02. The gamma parameter, a crucial component in SVC, was set within the range of {0,1} to facilitate the mapping of input datapoints into an infinite-dimensional space. Leveraging PCA, the feature dimensionality was effectively reduced, while SVC successfully classified the dataset into five distinct classes, as illustrated in (Fig. 5).

**Fig. 6:**
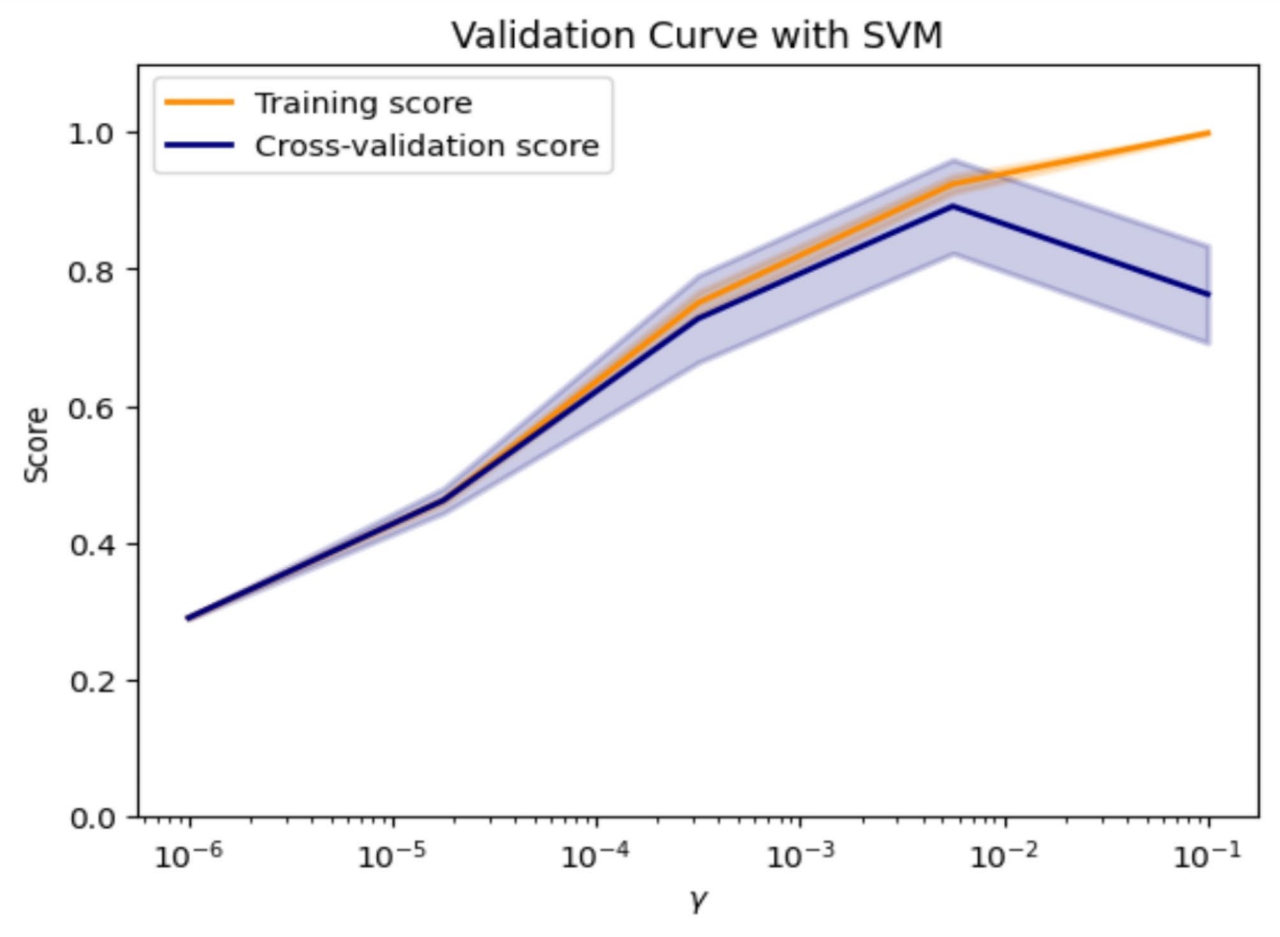
The validation curve of the SVC classifier.

**Fig. 7:**
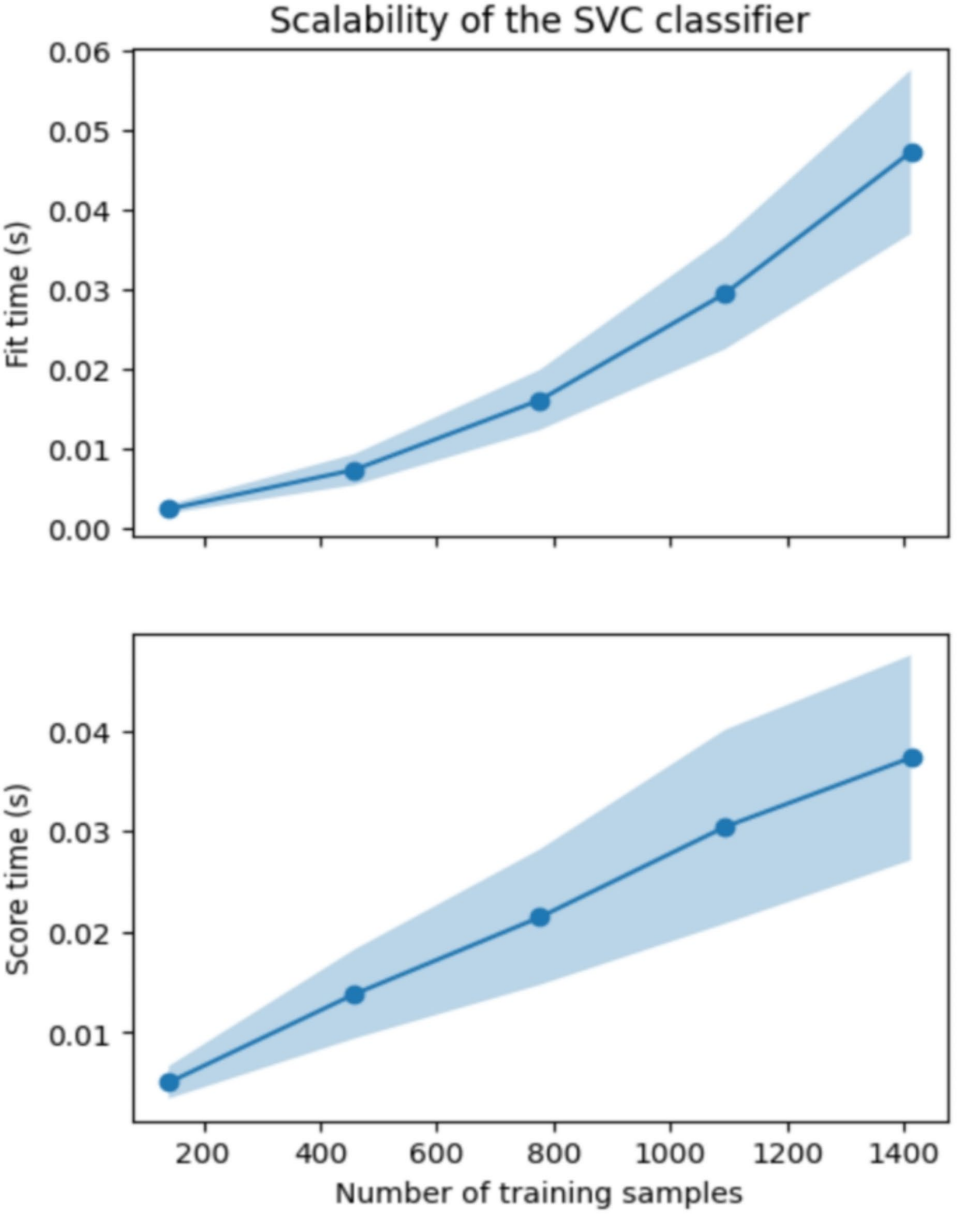
The validation curve of the SVC classifier.

Figure 6 illustrates the sensitivity of the model through a comparison between training, validation, and testing datasets, distributed in a 60:20:20 ratio following a stratification strategy to overcome imbalanced data distribution problem. Furthermore, Fig. 7 elucidates the scalability of the Support Vector Classifier (SVC) in terms of fit time (seconds) and score time (seconds) concerning varying numbers of training samples. Table 3 provides a comparative analysis between the Principal Component Analysis (PCA) coupled with SVC pipeline and alternative pipeline classifiers.

**Table 3:** Performance comparison between pipelined classifier models.

Pipeline models	Features	Accuracy (%)	Accuracy deviation	F1-Score (%)	Precision (%)	Recall (%)	MCC (%)
PCA + SVC (linear)	31	96	0.01	96	95	96	91
PCA + SVC (RBF)	34	92	0.02	92	91	92	86
PCA + Decision Tree (Gini)	18	75	0.03	75	74	76	71
PCA + Decision Tree (Entropy)	27	76	0.02	76	75	76	72
PCA + Naive Bayes	36	91	0.02	91	90	91	85
PCA + KNN	21	82	0.02	82	80	83	76

### Model explanations

**Fig. 8:**
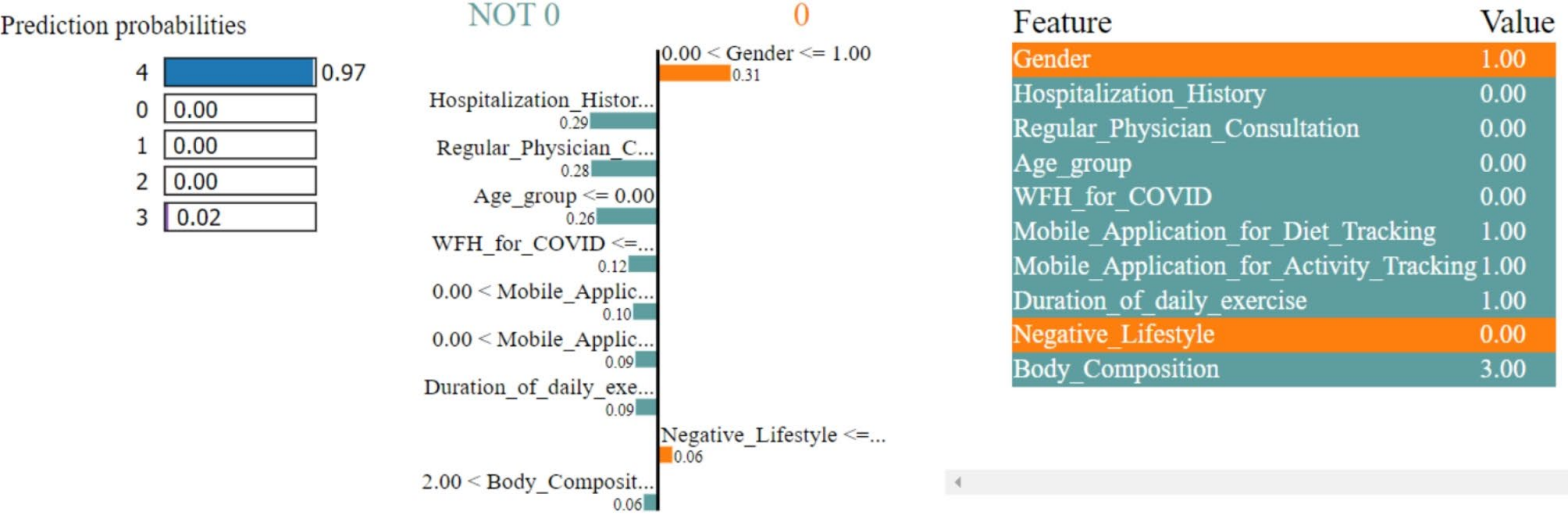
The LIME explanation notebook with top ten features for predicting class as 0.

**Fig. 9:**
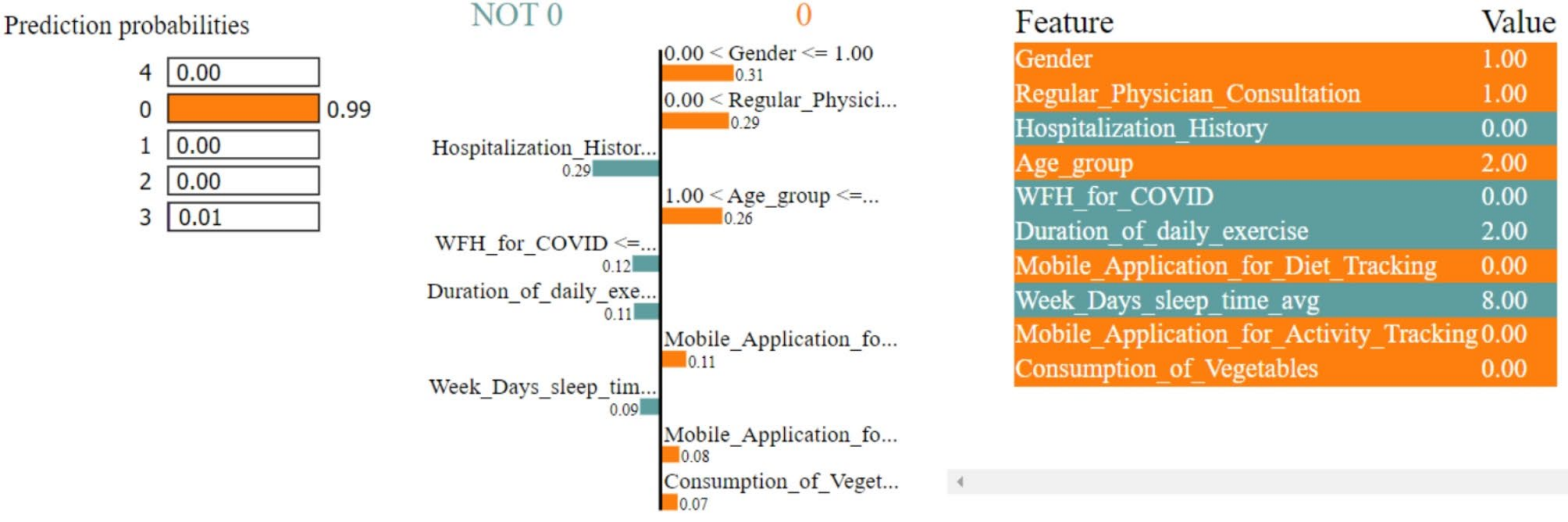
The LIME explanation notebook with top ten features for predicting class as 1.

**Fig. 10:**
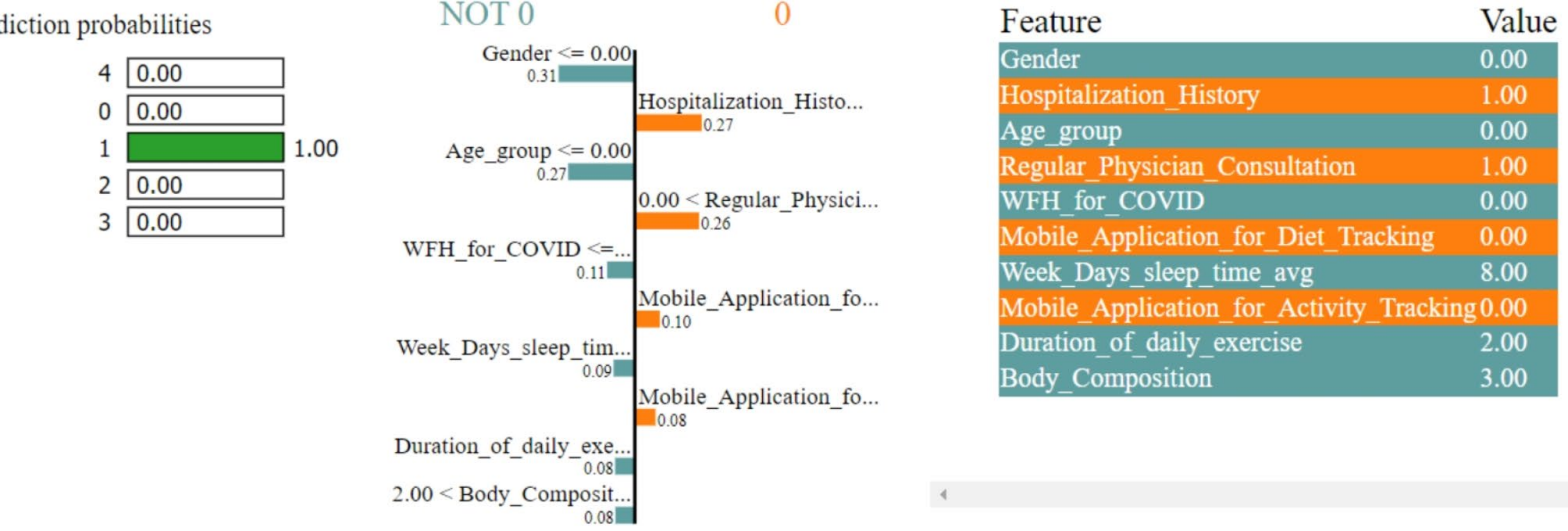
The LIME explanation notebook with top ten features for predicting class as 2.

**Fig. 11:**
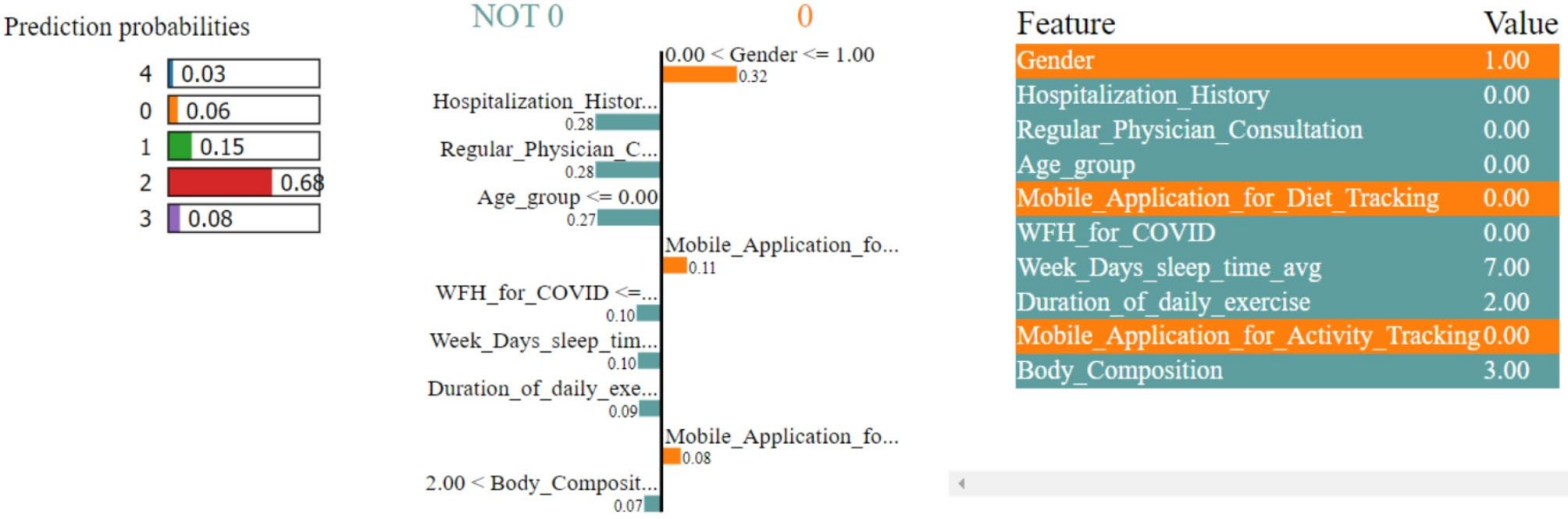
The LIME explanation notebook with top ten features for predicting class as 3.

**Fig. 12:**
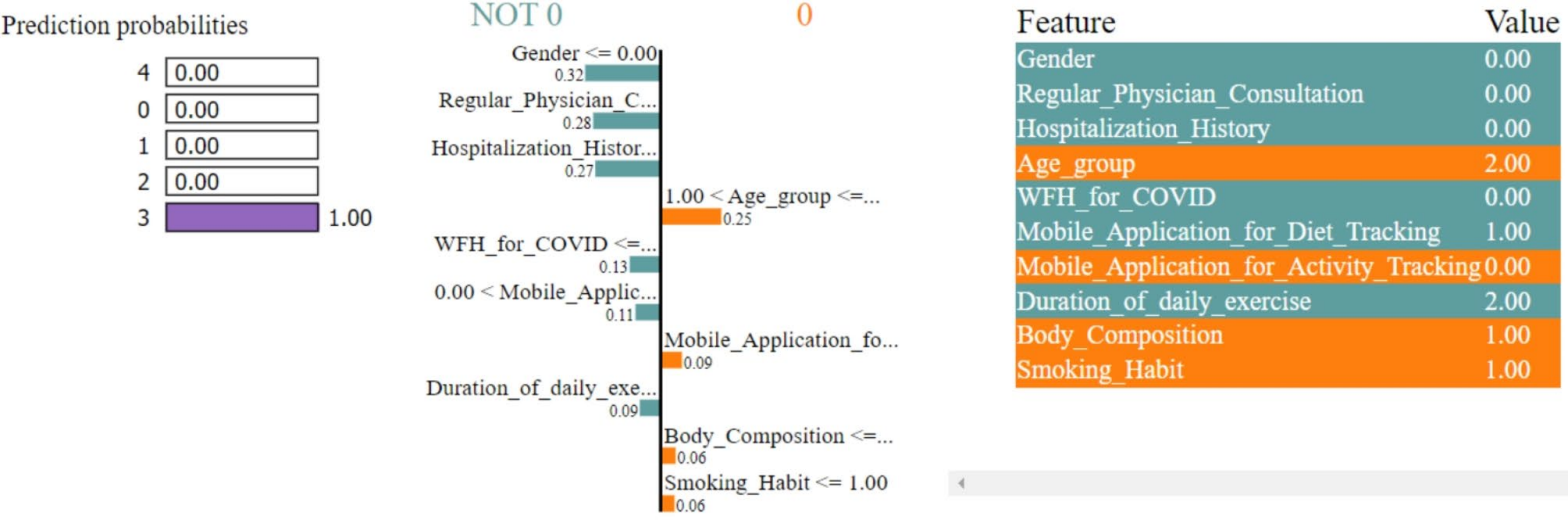
The LIME explanation notebook with top ten features for predicting class as 4.

In Figs. 8, 9, 10, 11 and 12, LIME presents the explanation in a tabular format, with rows representing different aspects of the explanation and columns providing details about each aspect. In this LIME-based multi-class prediction scenario, the colors serve to select possible outcome in the multi-class classification problem (e.g., Orange color represents high importance or influence of a feature for a particular class, Red indicates significant importance but slightly less than orange, Purple signifies moderate importance or influence of a feature on the prediction for a certain class, Green represents minimal importance or influence of a feature on the prediction for a particular class, and the Blue denotes negligible importance or influence of a feature for a given class). These colors help to visually interpret and understand the model’s predictions for individual instances.

In our LIME explanation table, the following columns are produced – (a) **Prediction Probabilities** to represent the predicted probabilities of each class provided by the model for the instance under consideration. In this predictive analysis task, where the goal is to classify instances into multiple classes, each row corresponds to a different class, and the prediction probability represents the likelihood of the instance belonging to that class according to the model, (b) **Classification** column indicates the class label associated with each row. It corresponds to the classes for which the prediction probabilities are provided. The class with the highest probability is typically chosen as the predicted class by the model, and (c) **Feature-Value** column displays the feature values for the instance being explained. Each row corresponds to a different feature, and the value represents the value of that feature for the instance under consideration. These feature values are typically normalized or scaled to ensure consistency across different features with varying scales or units. These columns collectively provide insights into how the model makes predictions for a specific instance (predict 0 or 1) and help to understand the features influencing the model’s decision. For the space limitation, we have considered the top ten features in the depiction of Figs. 8, 9, 10, 11 and 12.

### Semantic representation of knowledge

The questionnaire was structured into distinct categories encompassing personal, social, lifestyle, medical history, health status during the COVID-19 pandemic, and indicators of depression. Specifically, the lifestyle section was further subdivided into habit, dietary patterns, and physical activity.

**Fig. 13:**
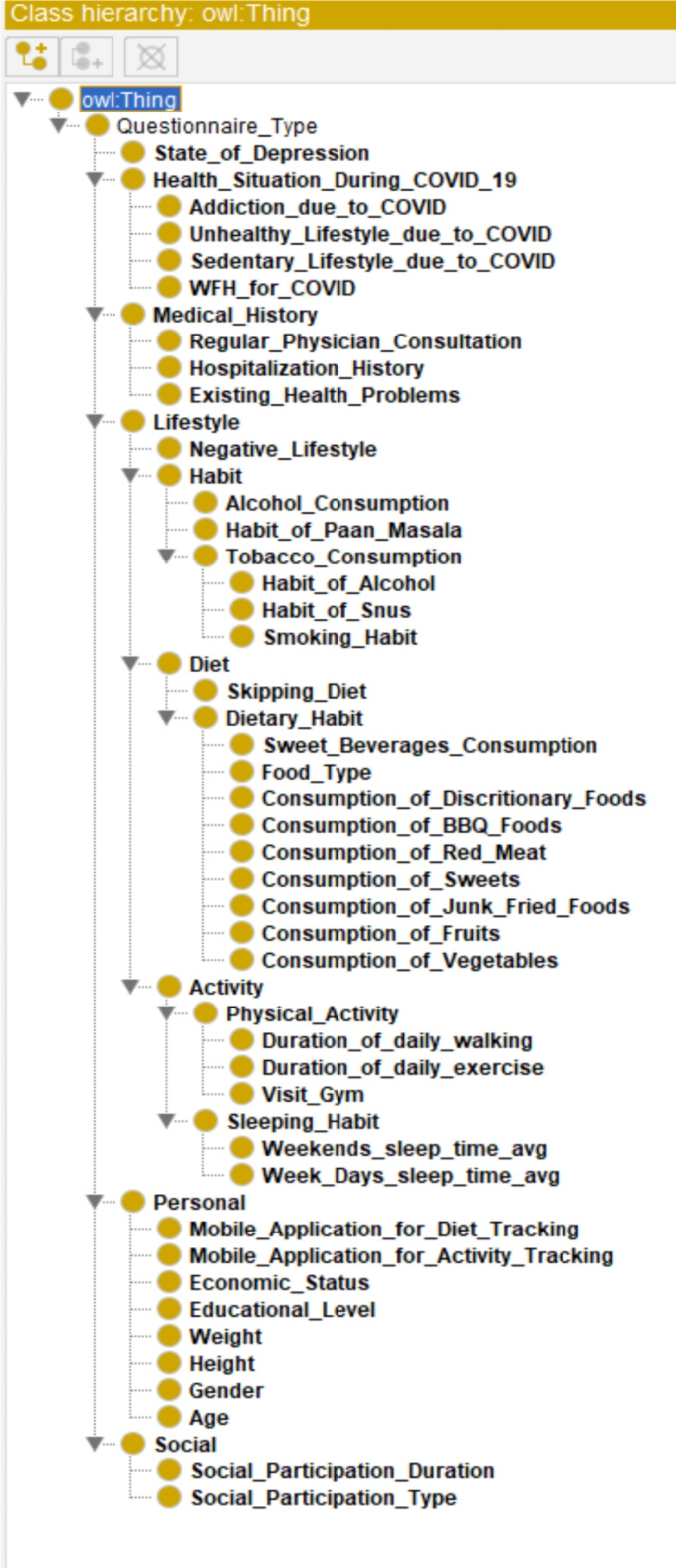
The asserted class hierarchy of the overall knowledge tree.

**Fig. 14:**
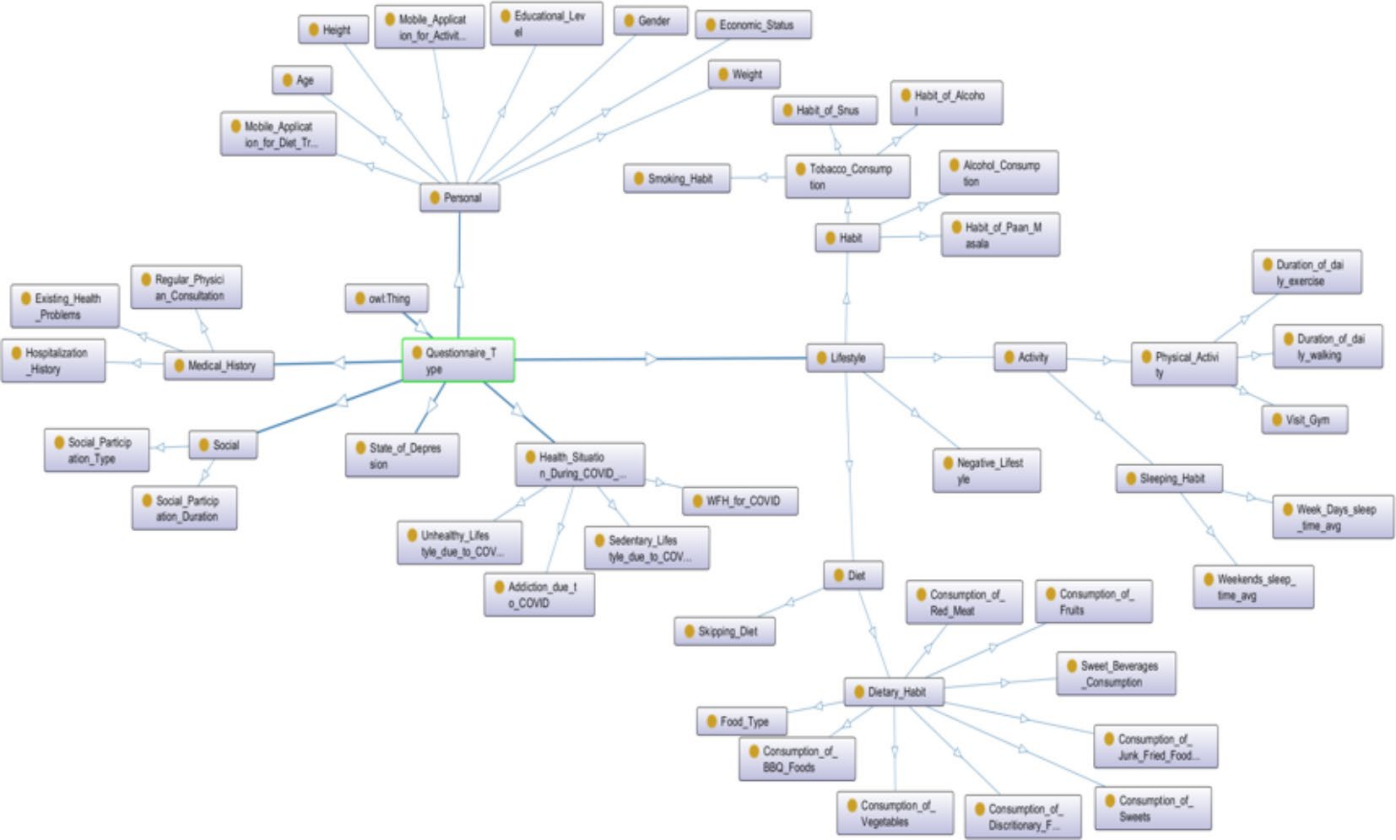
The logical structure of the ontology using OntoGraf.

To visually represent the comprehensive knowledge acquired, an OWL Ontology was constructed, utilizing an asserted class hierarchy (see Fig. 13). This Ontology comprised 103 Axioms, with 51 logical axioms and 52 declaration axioms. Notably, it included 52 distinct classes and 51 “Subclass Of” Class axioms. For ensuring structural integrity, the Hermit reasoner, integrated within Protégé 5, was employed to validate the Ontology. This validation process was completed within an approximate processing time of 2 s. Furthermore, the logical arrangement of the Ontology was elucidated through OntoGraf, as illustrated in (Fig. 14). 

## Discussion

**Fig. 15:**
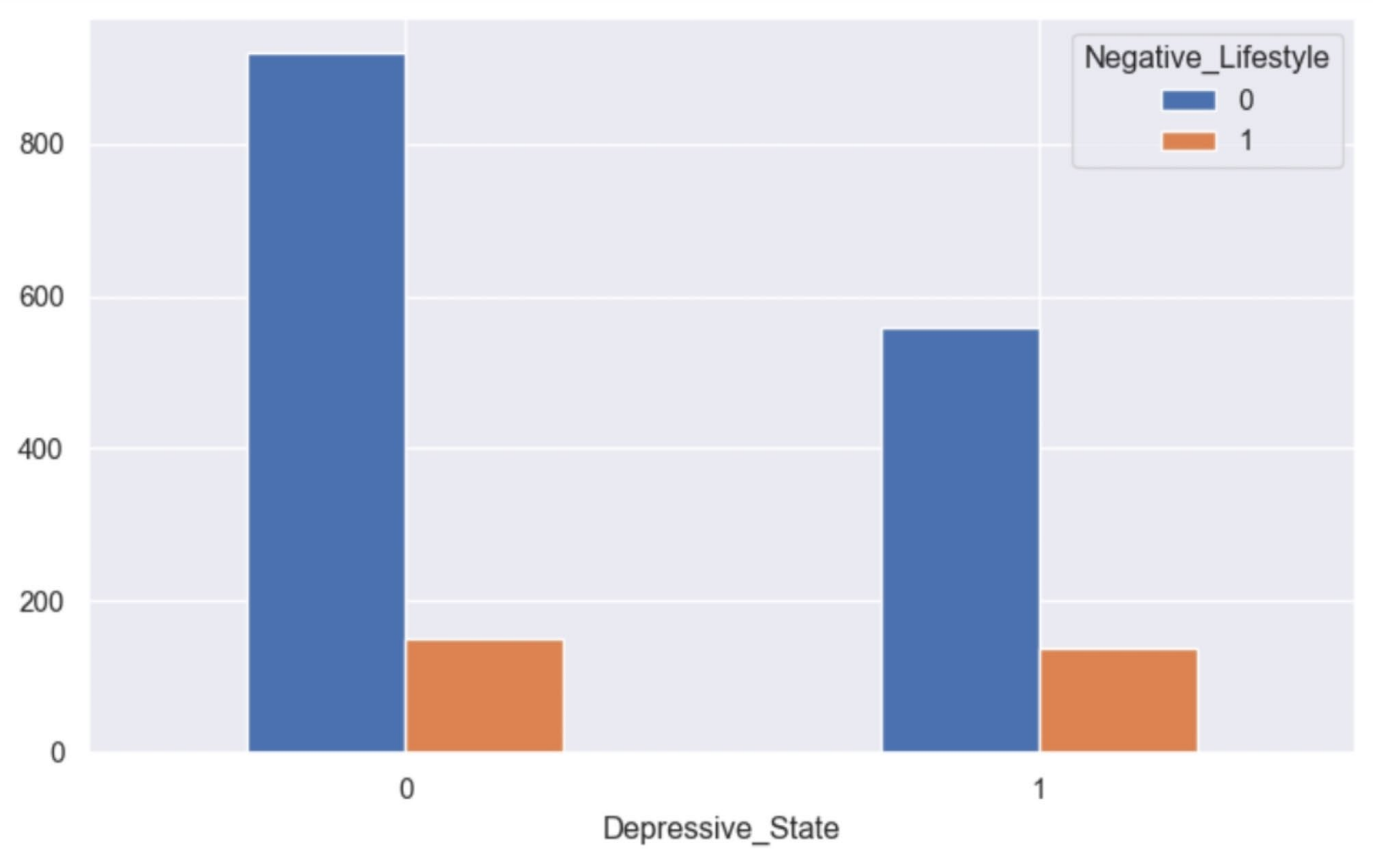
Grouped bar chart between negative lifestyle and depression.

Various factors play a role in the onset and perpetuation of depression. Adverse life events can heighten an individual’s vulnerability to depression or precipitate a depressive episode. Analysis conducted through the ANOVA test reveals that several factors exert a significant influence on depressive health among both genders. These factors encompass employment status, access to medical care, level of physical activity, sleep patterns, body composition, dietary habits, negative lifestyle patterns, irregular eating habits, substance dependencies, and remote work arrangements during the COVID-19 pandemic. Unhealthy lifestyle behaviors, such as poor dietary choices, reliance on sugary beverages and alcohol, tobacco consumption, insufficient sleep, and sedentary lifestyles, are implicated in the development and persistence of negative emotional states, including depression (see Fig. 15).

**Fig. 16:**
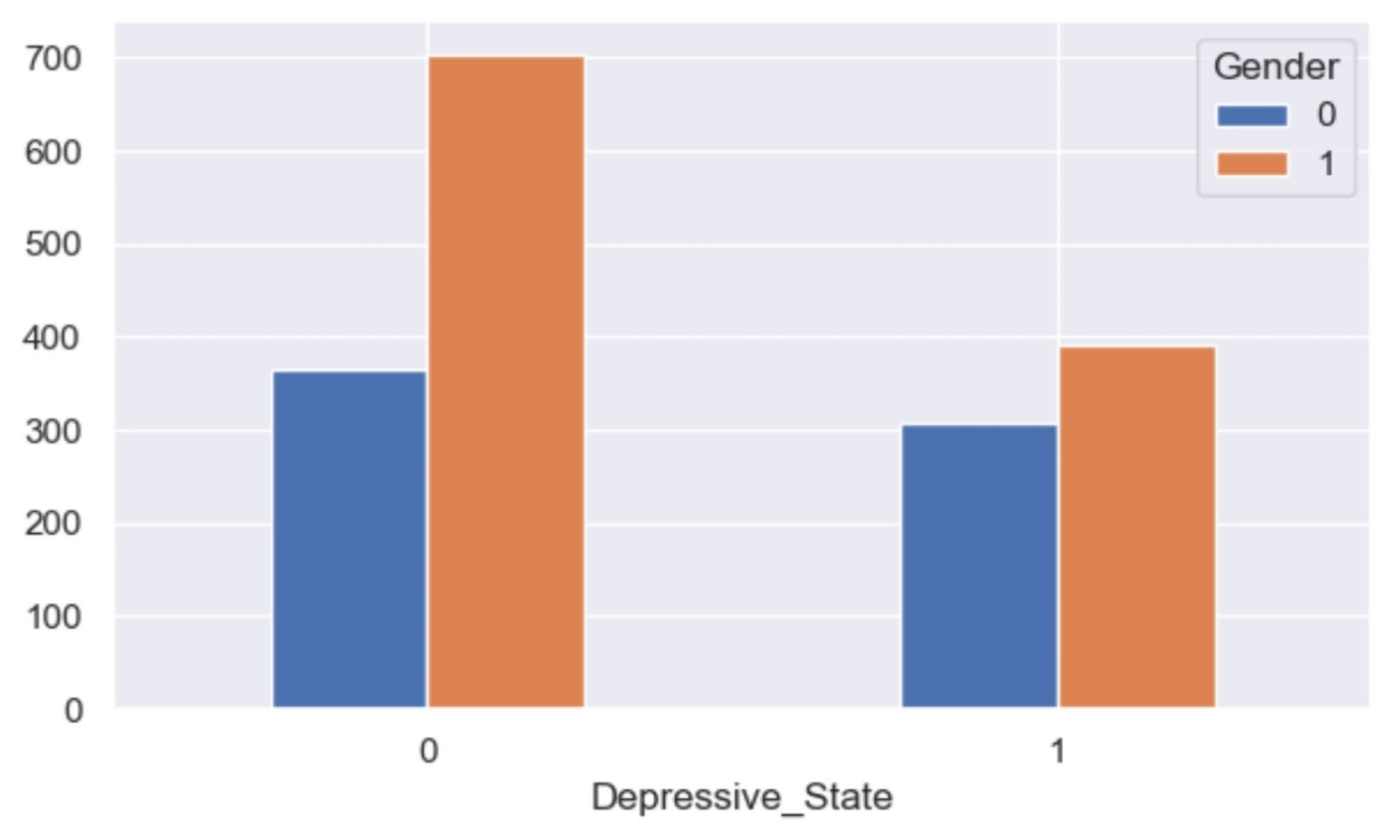
Grouped bar chart between gender and depression.

**Fig. 17:**
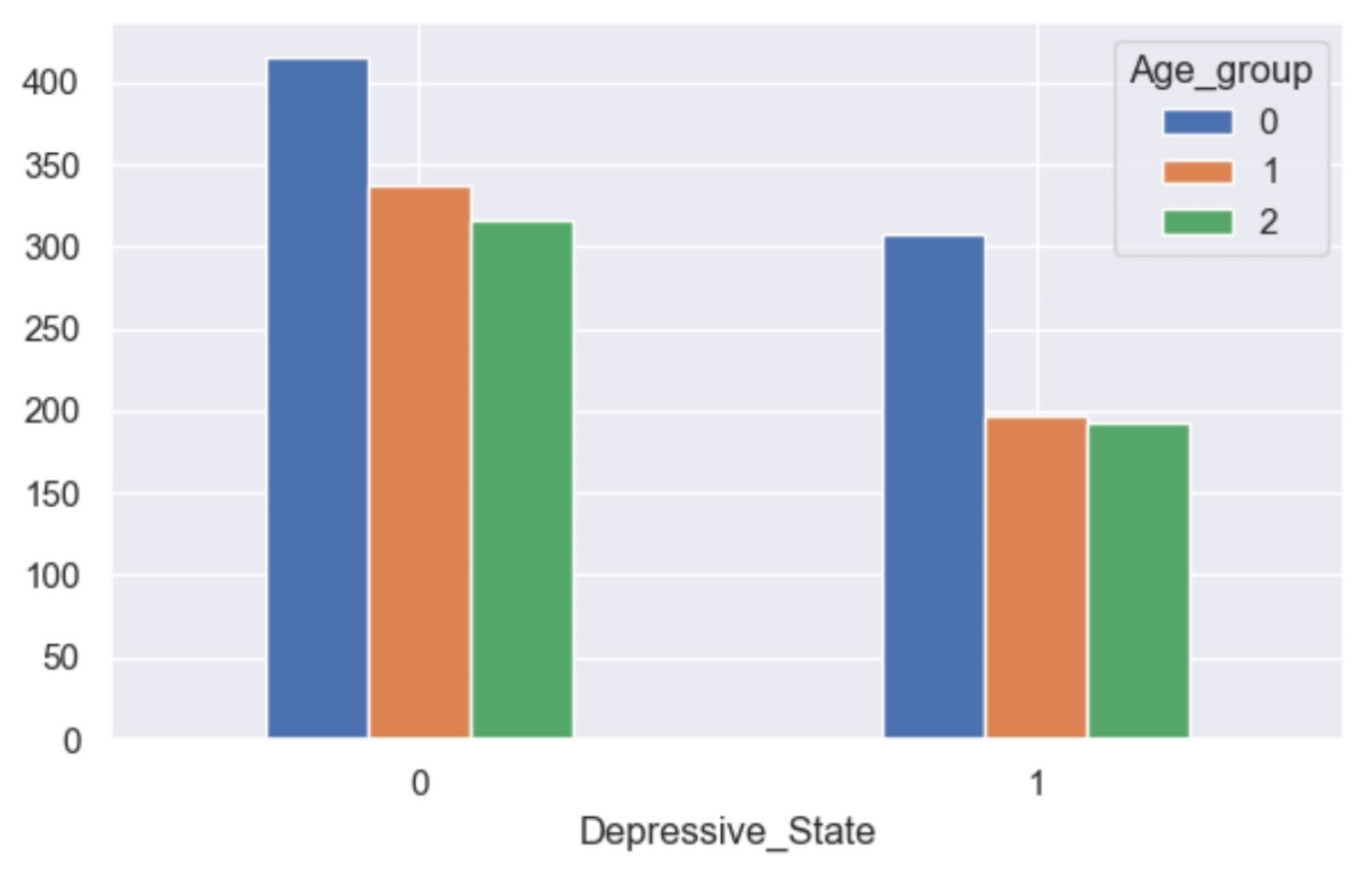
Grouped bar chart between age group and depression.

**Fig. 18:**
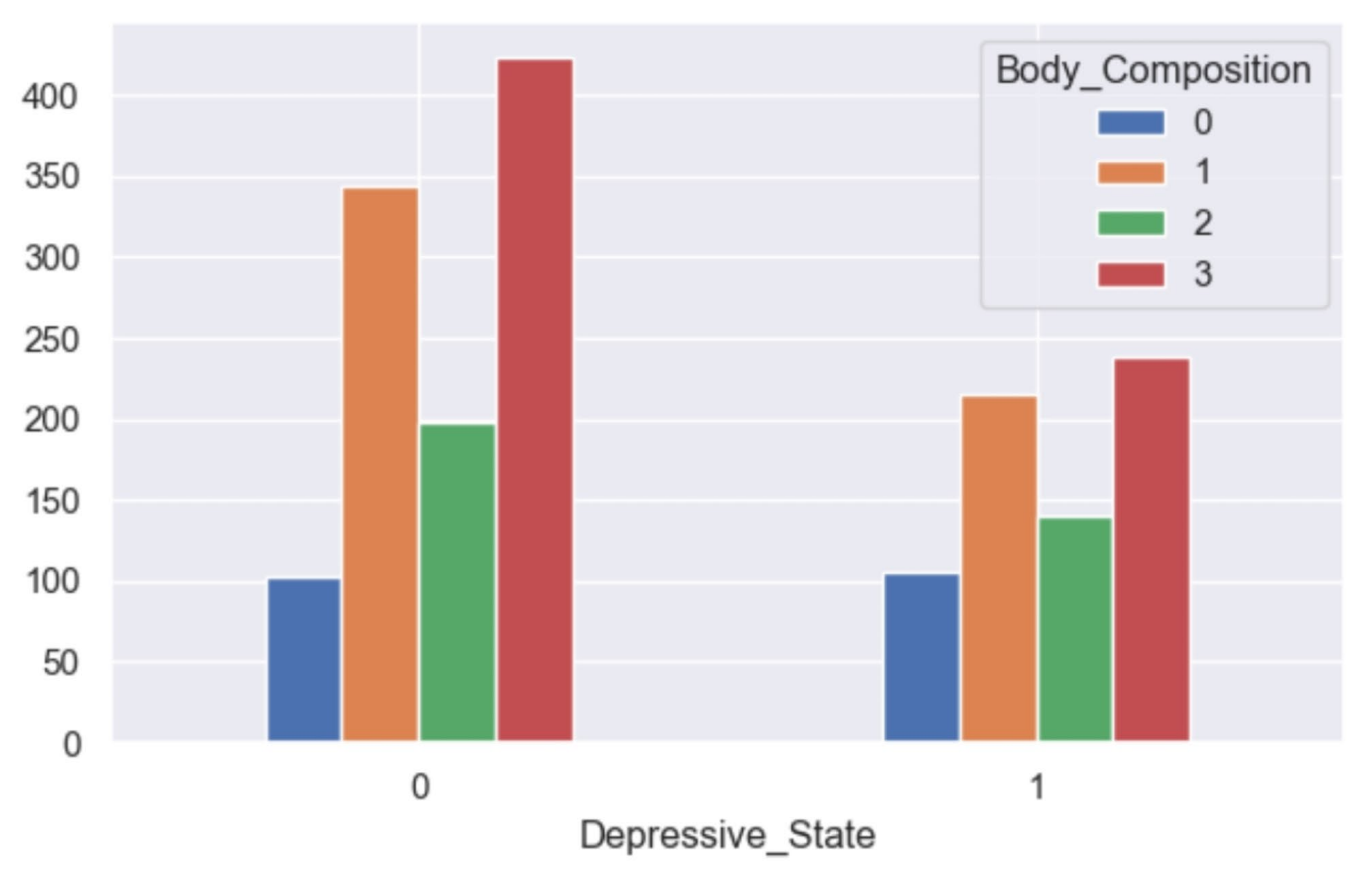
Grouped bar chart between body composition and depression.

The analysis reveals notable disparities in self-reported depression based on gender, with a higher prevalence observed among women (refer to Fig. 16). Similarly, age emerges as a significant factor, with older respondents exhibiting a greater likelihood of experiencing depression compared to their younger counterparts (refer to Fig. 17). Furthermore, individuals categorized as obese demonstrate a similarly elevated rate of depression (refer to Fig. 18). This aligns with the behavioral theory of depression^42,43^, which posits that avoidance behaviors contribute to the perpetuation of depressive symptoms. Individuals experiencing depression may engage in avoidance strategies, thereby exacerbating their condition. Therapeutic approaches informed by behavioral theory often incorporate interventions such as exposure and behavioral activation, which aim to encourage engagement in rewarding activities^44^. 

Utilizing K-Means clustering facilitated the identification of inherent patterns within the dataset and streamlined its scalability for enhanced convergence. This resulted in the formulation of a multi-class clustering problem, comprising five distinct classes, after dataset scaling. Employing a pipeline approach integrating principal component analysis (PCA) and conventional machine learning classifiers enabled the classification of the labeled dataset. PCA played a crucial role in reducing the dataset’s dimensionality, while machine learning classifiers effectively categorized the dataset using an optimized feature set. Notably, support vector classification (SVC) with a linear kernel emerged as the most effective classifier within the high-dimensional space. The OWL Ontology model has proven instrumental in facilitating queries utilizing the SPARQL Protocol and RDF Query Language (SPARQL), allowing for inquiries such as determining the number of participants who are both obese and experiencing depressive symptoms. Additionally, the ontology facilitates reasoning processes, such as utilizing height and weight measurements to calculate Body Mass Index (BMI) and subsequently grouping participants based on their body composition. This flexibility in ontology structure accommodates changes in survey dataset size, ensuring adaptability to evolving research requirements.

### Advantages, limitations, and future scope

Online surveys have emerged as a ubiquitous instrument across various research domains, including marketing and related disciplines. Yet, their utilization is increasingly prevalent within the realm of digital health. The potential advantages afforded by online surveys, as elucidated by the outcomes of this investigation, are succinctly delineated in Textbox 2.

**Textbox 2:** Advantages associated with the online public survey.

a. Easy data gathering – Most people are connected to social media, and they have smartphones with internet connectivity. Therefore, they can complete the questionnaire. b. Minimal cost – Traditional lifestyle data collection requires good infrastructure. However, online survey data collection is cheap as most people have smartphones with internet connections. c. Automated data input – When people are online, they can fill out the questionnaire and submit it based on their flexibility. It leads to hassle-free data handling with minimal errors. d. Good response rate – People can answer and submit the questionnaire based on their own pace, time, and preferences. e. Flexible questionnaire design – In an online survey, the Google Form supports a very flexible questionnaire set design with easy addition, deletion, and modification of questions. f. Accessibility from any device – People can submit their forms from any type of online device, such as a laptop, desktop, tablet, or mobile phone. g. Allows to be selective – The flexible questionnaire design in Google Forms allows the target population to be selective. h. Easy data visualization – The Google Form produces smart charts/visualization based on collected data and it helps in knowledge mining. i. Respondent Anonymity – The online survey using Google Forms can maintain anonymity during data collection. j. No interviewer – As our online survey requires no interviewer, people can answer the questions with more freedom and flexibility without the fear of identity disclosure.	

The utilization of online surveys, while convenient, often presents inherent challenges. Despite Kolkata’s significant population density, our survey yielded a relatively limited dataset. The constraints of this online public survey are delineated in (Textbox 3). Through careful design of the questionnaire implemented in the Google Form platform, we mitigated issues such as response duplication and survey fraud, exemplified by instances of question skipping. In forthcoming investigations, we aim to explore the integration of sentiment analysis methodologies to enhance our understanding of respondent motivations and attitudes. Most of the existing Expressed and private opinions (EPOs) models assumed that the individual private opinion is often fixed or updated by his/her own private opinion and neighbors’ expressed opinions, ignoring the behavior characteristics of social individuals^45^.

**Textbox 3:** Limitations associated with the online public survey.

a. Willingness – People can understand the importance of the survey; however, they are either not motivated or willing to submit their views/feedback/answers. b. Language problem – Many people in Kolkata are more flexible in regional languages than the English. c. Doubts – As there are no trained interviewers present in this survey with open-ended questions, many people faced doubts; but, feared to approach. d. Difficulty in reaching challenging population – We failed to reach many challenging participants from remote areas due to their lack of internet connectivity and social media reluctance. e. Lack of rewards – Many participants were reluctant to submit their answers as the participation was voluntary. f. Survey fatigue – Some participants dropped out due to unwillingness to answer 44 questions. g. Dishonest response – Some participants provided incorrect data in their response, and they were identified as outliers. h. Digital literacy – Many participants were able to use smartphones in terms of attending general calls, general messaging and browsing; however, they were not confident in participating in online survey. i. Sampling error – Though we targeted population with age > = 18 and < 65, many participants outside of the age group participated in the survey. We deleted such records.	

While the study presents a robust methodology for investigating the relationship between lifestyle and demographic factors and depressive symptoms during the COVID-19 pandemic, several limitations should be acknowledged (see Textbox 4). The study acknowledges advantages such as anonymity and accessibility; however, potential ethical concerns related to data privacy, informed consent, and protection of participant confidentiality should be carefully addressed to ensure ethical research conduct. Addressing these limitations in future research endeavors could enhance the robustness and validity of findings, ultimately contributing to a deeper understanding of the complex interplay between lifestyle factors, demographic characteristics, and depressive symptoms during the COVID-19 pandemic.

**Textbox 4:** Limitations of this study.

a. Sampling Bias – The study’s reliance on an online survey may introduce sampling bias, as it excludes individuals without internet access or digital literacy. This could lead to underrepresentation of certain demographic groups, potentially skewing the results. b. Self-Reporting Bias – The data collected through self-reported measures may be subject to bias, as participants may provide inaccurate or incomplete information due to social desirability or recall biases. This could affect the reliability and validity of the findings. c. Limited Generalizability – Conducting the study in Kolkata, India, may limit the generalizability of the findings to other regions or populations with different socio-cultural contexts. The study’s focus on a specific geographic area may restrict the applicability of the results to broader populations. d. Data Quality and Noise – Despite efforts to clean the dataset by removing missing data and outliers, the presence of residual noise or errors may still impact the validity of the results. Without robust measures to address data quality issues, the accuracy of the findings may be compromised. e. Lack of Longitudinal Data - The study’s cross-sectional design and reliance on data collected at a single point in time limit its ability to capture the dynamic nature of depressive symptoms over time, hindering a comprehensive understanding of the longitudinal impact of lifestyle and demographic factors on mental health outcomes. f. Language and Digital Literacy Barriers - The use of online surveys conducted in English may exclude individuals who are not proficient in the language or lack digital literacy skills, potentially leading to underrepresentation of certain demographic groups and limiting the inclusivity and diversity of the sample. g. Methodological Constraints – While the study employs advanced statistical and machine learning techniques, such as PCA and SVC, other relevant methods or approaches may not have been considered. The omission of alternative methodologies could limit the comprehensiveness of the analysis and interpretation of the results. h. Ontology Development – While the use of an OWL Ontology facilitates semantic representation of the dataset, challenges in ontology development, such as ontology alignment, scalability, and maintenance, may affect the utility and usability of the semantic model.	

This study provides valuable insights into the intricate interplay among lifestyle factors, demographic characteristics, and symptoms of depression during the COVID-19 pandemic. However, it is essential to acknowledge the inherent uncertainties in such research endeavors. One primary uncertainty lies in the generalizability of findings, given that the studies predominantly focus on specific populations, such as Indians in Kolkata, India. This limitation raises questions about the broader applicability of the results to other cultural or geographical contexts. Additionally, the reliance on online surveys introduces sampling biases, as certain demographic groups may be underrepresented or excluded, thereby impacting the representativeness of the data.

Moreover, while the methodologies employed are comprehensive, they may overlook nuanced aspects of depression or lifestyle factors due to the complexity of these constructs. For instance, the use of machine learning algorithms for classification may yield high accuracy rates but could potentially overlook important contextual nuances or confounding variables. Furthermore, the challenges highlighted in the studies, such as language barriers and digital literacy issues, underscore the importance of carefully considering data quality and inclusivity of participants. Despite these uncertainties, the studies collectively contribute to our understanding of depression risk factors and provide valuable insights into addressing mental health challenges through scalable approaches like online public surveys. However, future research should aim to address these uncertainties by employing diverse methodologies, expanding sample diversity, and considering broader contextual factors to enhance the robustness and generalizability of findings.

## Conclusion

This study comprises five main components. Firstly, amidst the second wave of COVID-19 in Kolkata, India, we conducted an extensive three-month online questionnaire-based public survey aimed at gathering demographic and lifestyle data from participants. Subsequently, the datasets underwent thorough statistical analysis in the second phase to identify factors potentially linked to depressive health among the respondents. In the third phase, we applied clustering techniques to the unlabeled dataset to delineate inherent feature patterns and assign appropriate labels. Next, to optimize dataset classification, we conducted predictive analyses using PCA in conjunction with standard machine learning algorithms. Finally, we developed an OWL Ontology to semantically represent the questionnaire, enhancing comprehension of the gathered data. Furthermore, we provided a comprehensive discussion on the advantages and limitations of using online surveys in research. Additionally, all acquired data have been made publicly accessible to facilitate further investigation by fellow researchers.

## Electronic supplementary material

Below is the link to the electronic supplementary material.

Supplementary Material 1

Supplementary Material 2

Supplementary Material 3

Supplementary Material 4

## Data Availability

All the data used or produced in this study are either in the main text or in the supplementary files. The GitHub codebase has been made publicly available. The corresponding author **AC** can be contacted for further information. GitHub: https://github.com/ayan1c2/Survey_COVID_Lifestyle.git.

## Abbreviations

ML: Machine learning
PCA: Principal component analysis
OWL: Web ontology language
ANOVA: Analysis of variance
KNN: K-Nearest neighbor
SVC: Support vector classifier
NB: Naïve bayes
StaRI: Standards for reporting implementation
RQ: Research question
SPARQL: SPARQL protocol and RDF query language
WWW: World wide web
GDPR: General data protection regulation
SIKT: Norwegian agency for shared services in education and research
EPOs: Expressed and private opinions

## References

[1] Yang, S. et al. The use of ICT during COVID-19. *Proc. Assoc. Inf. Sci. Technol.*** 57**(1), e297. 10.1002/pra2.297 (2020). 33173819 10.1002/pra2.297 PMC7645918

[2] Oliver, J., Dutch, M., Rojek, A., Putland, M. & Knott, J. C. Remote COVID-19 patient monitoring system: a qualitative evaluation. *BMJ Open*** 12**(5), e054601 (2022). 10.1136/bmjopen-2021-054601 PMC9072784 35508350

[3] Chatterjee, A., Gerdes, M. W. & Martinez, S. G. Statistical explorations and univariate timeseries analysis on COVID-19 datasets to understand the trend of disease spreading and death. *Sensors*** 20**(11), 3089 (2020). 32486055 10.3390/s20113089 PMC7308840

[4] Pronovost, P. J., Cole, M. D. & Hughes, R. M. Remote patient monitoring during COVID-19: an unexpected patient Safety Benefit. *JAMA*** 327**(12), 1125–1126 (2022). 35212725 10.1001/jama.2022.2040

[5] Everyone Included. Social Impact of COVID-19. https://www.un.org/development/desa/dspd/everyone-included-covid-19.html (2023).

[6] COVID-19 pandemic. triggers 25% increase in prevalence of anxiety and depression worldwide. https://www.who.int/news/item/02-03-2022-covid-19-pandemic-triggers-25-increase-in-prevalence-of-anxiety-and-depression-worldwide (2023). PMC9998057 35414629

[7] COVID-19 in India. https://covid19.who.int/region/searo/country/in (2023).

[8] Roy, D. et al. Study of knowledge, attitude, anxiety & perceived mental healthcare need in Indian population during COVID-19 pandemic. *Asian J. Psychiatr.*** 51**, 102083. 10.1016/j.ajp.2020.102083 (2020). 10.1016/j.ajp.2020.102083 PMC7139237 32283510

[9] Daly, M. & Robinson, E. Depression and anxiety during COVID-19. *Lancet*** 399**(10324), 518 (2022). 35123689 10.1016/S0140-6736(22)00187-8 PMC8813060

[10] Depression January. https://www.who.int/news-room/fact-sheets/detail/depression (2023).

[11] Depression (major depressive disorder). https://www.mayoclinic.org/diseases-conditions/depression/symptoms-causes/syc-20356007 (2023).

[12] COVID-19 and Depression. https://www.webmd.com/covid/covid-19-depression (2023).

[13] Santomauro, D. F., Herrera, A. M. M., Shadid, J., Zheng, P., Ashbaugh, C., Pigott,D. M., Ferrari, A. J. Global prevalence and burden of depressive and anxiety disorders in 204 countries and territories in 2020 due to the COVID-19 pandemic. *Lancet*** 398**(10312), 1700–1712. (2021). 10.1016/S0140-6736(21)02143-7 PMC8500697 34634250

[14] Giuntella, O., Hyde, K., Saccardo, S. & Sadoff, S. Lifestyle and mental health disruptions during COVID-19. *Proc. Natl. Acad. Sci.*** 118**(9), e2016632118 (2021). 33571107 10.1073/pnas.2016632118 PMC7936339

[15] Rawat, D., Dixit, V., Gulati, S., Gulati, S. & Gulati, A. Impact of COVID-19 outbreak on lifestyle behaviour: a review of studies published in India. *Diabetes Metabolic Syndrome: Clin. Res. Rev.*** 15**(1), 331–336 (2021). 10.1016/j.dsx.2020.12.038 PMC7837201 33493852

[16] Yun, J. Y. et al. Impact of COVID-19 on lifestyle, personal attitudes, and mental health among Korean medical students: network analysis of associated patterns. *Front. Psychiatr.* 12. (2021). 10.3389/fpsyt.2021.702092 PMC8416342 34483994

[17] Singh, B., Jain, S. & Rastogi, A. Effects of nationwide COVID-19 lockdown on lifestyle and diet: an Indian survey. *J. Family Med. Prim. Care*** 10**(3), 1246 (2021). 34041159 10.4103/jfmpc.jfmpc_2046_20 PMC8140248

[18] Dolton, P. The statistical challenges of modelling COVID-19. *Natl. Inst. Econ. Rev.*** 257**, 46–82 (2021).

[19] Welte, T. et al. Current evidence for COVID-19 therapies: a systematic literature review. *Eur. Respiratory Rev.*** 30**(159), (2021). 10.1183/16000617.0384-2020 PMC9489065 33731328

[20] Surkalim, D. L. et al. The prevalence of loneliness across 113 countries: systematic review and meta-analysis. *Bmj* 376, (2022). 10.1136/bmj-2021-067068 PMC8826180 35140066

[21] Vehovar, V. & Manfreda, K. L. Overview: online surveys. *SAGE Handb. Online Res. Methods*** 1**, 177–194 (2008).

[22] Dillman, D. A. & Bowker, D. K. The web questionnaire challenge to survey methodologists. *Online Soc. Sci.*** 7**, 53–71 (2001).

[23] Van Selm, M. & Jankowski, N. W. Conducting online surveys. *Qual. Quantity*** 40**(3), 435–456 (2006).

[24] Ball, H. L. Conducting online surveys. *J. Hum. Lactation*** 35** (3), 413–417 (2019). 10.1177/0890334419848734 31084575

[25] Lehdonvirta, V., Oksanen, A., Räsänen, P. & Blank, G. Social media, web, and panel surveys: using non-probability samples in social and policy research. *Policy Internet*** 13**(1), 134–155 (2021).

[26] Schneider, D. & Harknett, K. What’s to like? Facebook as a tool for survey data collection. *Sociol. Methods Res.*** 51**(1), 108–140 (2022). 36845408 10.1177/0049124119882477 PMC9957582

[27] Chatterjee, A., Gerdes, M. W. & Martinez, S. G. Identification of risk factors associated with obesity and overweight—A machine learning overview. *Sensors*** 20**(9), 2734 (2020). 32403349 10.3390/s20092734 PMC7248873

[28] Myers, L. & Sirois, M. J. *Spearman Correlation Coefficients, Differences between*12 (Encyclopedia of statistical sciences, 2004).

[29] Cuevas, A., Febrero, M. & Fraiman, R. An anova test for functional data. *Comput. Stat. Data Anal.*** 47**(1), 111–122 (2004).

[30] Likas, A., Vlassis, N. & Verbeek, J. J. The global k-means clustering algorithm. *Pattern Recogn.*** 36**(2), 451–461 (2003).

[31] Lovmar, L., Ahlford, A., Jonsson, M. & Syvänen, A. C. Silhouette scores for assessment of SNP genotype clusters. *BMC Genom.*** 6**(1), 1–6 (2005). 10.1186/1471-2164-6-35 PMC555759 15760469

[32] Syakur, M. A., Khotimah, B. K., Rochman, E. M. S. & Satoto, B. D. Integration k-means clustering method and elbow method for identification of the best customer profile cluster. In: *IOP conference series: materials science and engineering*** 336**(1), 012017. (IOP Publishing, 2018).

[33] Chatterjee, A., Gerdes, M. W., Prinz, A. & Martinez, S. G. Comparing performance of Ensemble-Based Machine Learning Algorithms to identify potential obesity risk factors from Public Health datasets. In emerging technologies in data mining and information security (253–269). (Springer, 2021).

[34] Chatterjee, A., Pahari, N., Prinz, A. & Riegler, M. Machine learning and ontology in eCoaching for personalized activity level monitoring and recommendation generation. *Sci. Rep.*** 12**(1), 1–26 (2022). 36400793 10.1038/s41598-022-24118-4 PMC9674665

[35] Lee, D. & Lee, J. Domain described support vector classifier for multi-classification problems. *Pattern Recogn.*** 40**(1), 41–51 (2007).

[36] Quinlan, J. R. Learning decision tree classifiers. *ACM Comput. Surv. (CSUR)*** 28**(1), 71–72 (1996).

[37] Chatterjee, A., Gerdes, M. W., Prinz, A. & Martinez, S. A comparative study to analyze the performance of advanced pattern recognition algorithms for multi-class classification. In: Emerging Technologies in Data Mining and Information Security (111–124). (Springer, 2021).

[38] Guo, G., Wang, H., Bell, D., Bi, Y. & Greer, K. KNN model-based approach in classification. In OTM Confederated International Conferences On the Move to Meaningful Internet Systems (986–996). (Springer, 2003)

[39] Garreau, D. & Luxburg, U. Explaining the explainer: A first theoretical analysis of LIME. In International conference on artificial intelligence and statistics (1287–1296). (PMLR, 2020).

[40] Chatterjee, A., Prinz, A., Gerdes, M. & Martinez, S. An automatic ontology-based approach to support logical representation of observable and measurable data for healthy lifestyle management: Proof-of-concept study. *J. Med. Internet. Res.*** 23**(4), e24656. (2021). 10.2196/24656 PMC8065560 33835031

[41] Chatterjee, A. & Prinz, A. Personalized recommendations for physical activity e-Coaching (OntoRecoModel): Ontological modeling. *JMIR Med. Inf.*** 10**(6), e33847. (2022). 10.2196/33847 PMC9282669 35737439

[42] Lewinsohn, P. M. & Gotlib, I. H. Behavioral theory and treatment of depression. (1995).

[43] Carvalho, J. P. & Hopko, D. R. Behavioral theory of depression: reinforcement as a mediating variable between avoidance and depression. *J. Behav. Ther. Exp. Psychiatr.*** 42**(2), 154–162 (2011). 21315876 10.1016/j.jbtep.2010.10.001

[44] Steven, D., Hollon, M. O., Stewart & Strunk, D. Enduring effects for Cognitive Behavior Therapy in the treatment of depression and anxiety. *Ann. Rev. Psychol.*** 57**(1), 285–315. (2006). 10.1146/annurev.psych.57.102904.190044 16318597

[45] Dong, J., Hu, J., Zhao, Y. & Peng, Y. Opinion formation analysis for expressed and private opinions (EPOs) models: reasoning private opinions from behaviors in group decision-making systems. *Expert Syst. Appl.*** 236**, 121292 (2024).

