# Author Correction: Exploring online public survey lifestyle datasets with statistical analysis, machine learning and semantic ontology

**DOI:** 10.1038/s41598-025-86904-0

**Published:** 2025-02-10

**Authors:** Ayan Chatterjee, Michael A. Riegler, Miriam Sinkerud Johnson, Jishnu Das, Nibedita Pahari, Raghavendra Ramachandra, Bikramaditya Ghosh, Arpan Saha, Ram Bajpai

**Affiliations:** 1 Department of Information and Communication Technologies, University of Agder, 4879 Grimstad, Norway; 2 Department of Holistic Systems, Simula Metropolitan Center for Digital Engineering, 0167 Oslo, Norway; 3 Department of Digital Technology, STIFTELSEN NILU, 2007 Kjeller, Norway; 4 Department of Behavioral Sciences, Oslo Metropolitan University, 0176 Oslo, Norway; 5 Department of Information Systems, University of Agder, 4630 Kristiansand, Norway; 6 Department of Software Development, Maulana Abul Kalam Azad University of Technology, Kolkata, 700064 India; 7 Department of Information Security and Communication Technology, Norwegian University of Science and Technology, 2815 Gjøvik, Norway; 8 Department of Finance, Symbiosis Institute of Business Management, Symbiosis International (Deemed University), Bengaluru, 560100 India; 9 Department of Finance, Harper Adams University, Shropshire, TF10 8NB UK; 10 Department of Pharmaceutical Chemistry, Bharat Pharmaceutical Technology, Tripura, 799130 India; 11 School of Medicine, Keele University, Keele, Newcastle, ST5 5BG UK

Correction to: *Scientific Reports* 10.1038/s41598-024-74539-6, published online 15 October 2024

In the original version of this Article Michael A. Riegler was incorrectly affiliated with the ‘Department of Digital Technology, STIFTELSEN NILU, 2007, Kjeller, Norway’. The correct affiliation is listed below:

Department of Holistic Systems, Simula Metropolitan Center for Digital Engineering, 0167, Oslo, Norway

The original Article has been corrected.

