# Supplementary material for: Exploring online public survey lifestyle datasets with statistical analysis, machine learning and semantic ontology
Source: Sci Rep. 2024 Oct 15;14:24190. doi: 10.1038/s41598-024-74539-6 (PMC11480510; doi:10.1038/s41598-024-74539-6)
Supplement: Supplementary file 1 — Supplementary Material 1 [file 41598_2024_74539_MOESM1_ESM.docx]

**Table S3.1**. The description of the asked questions based on n=1,767 responses.

| **Feature name** | **Type** | **Unit** | **Mean** | **Std** | **25%** | **50%** | **75%** |
| --- | --- | --- | --- | --- | --- | --- | --- |
| Gender | Categorical | - | 0.620260 | 0.485459 | 0 | 1 | 1 |
| Dependant | Non-categorical | - | 2.099038 | 1.086223 | 1 | 2 | 3 |
| Is_Employed | Categorical | - | 4.161290 | 2.753052 | 2 | 4 | 7 |
| Is_Medically_Insured | Categorical | - | 0.831919 | 0.374044 | 1 | 1 | 1 |
| Visit_Gym | Categorical | - | 0.203735 | 0.402888 | 0 | 0 | 0 |
| Week_Days_sleep_time_avg | Non-categorical | Mins | 8.495430 | 1.145373 | 8 | 9 | 9 |
| Weekends_sleep_time_avg | Non-categorical | Mins | 8.687889 | 1.388478 | 8 | 9 | 9 |
| Age_group | Categorical | - | 0.878325 | 0.826665 | 0 | 1 | 2 |
| Body_Composition | Categorical | - | 1.822864 | 1.063541 | 1 | 2 | 3 |
| Educational_Level | Categorical | - | 3.046406 | 1.474240 | 2 | 2 | 5 |
| Economic_Status | Categorical | - | 1.434069 | 1.353253 | 1 | 1 | 1 |
| Duration_of_daily_exercise | Categorical | Mins | 1.745331 | 0.829591 | 2 | 2 | 2 |
| Duration_of_daily_walking | Categorical | Mins | 0.378041 | 0.902201 | 0 | 0 | 0 |
| Smoking_Habit | Categorical | - | 1.091680 | 0.495247 | 1 | 1 | 1 |
| Habit_of_Snus | Categorical | - | 0.026598 | 0.160953 | 0 | 0 | 0 |
| Habit_of_Paan_Masala | Categorical | - | 1.492925 | 0.723204 | 1 | 2 | 2 |
| Habit_of_Alcohol | Categorical | - | 1.308432 | 0.547259 | 1 | 1 | 2 |
| Habit_of_Energy_Drinks | Categorical | - | 1.492925 | 0.723204 | 1 | 2 | 2 |
| Mobile_Application_for_Activity_Tracking | Categorical | - | 0.307866 | 0.461741 | 0 | 1 | 1 |
| Mobile_Application_for_Diet_Tracking | Categorical | - | 0.311262 | 0.463140 | 0 | 0 | 1 |
| Social_Participation_Type | Categorical | - | 0.676853 | 0.6307 | 0 | 1 | 1 |
| Social_Participation_Duration | Categorical | - | 0.448217 | 0.497452 | 0 | 0 | 1 |
| Consumption_of_Vegetables | Categorical | - | 0.028296 | 0.233862 | 0 | 0 | 0 |
| Consumption_of_Fruits | Categorical | - | 0.134125 | 0.498702 | 0 | 0 | 0 |
| Consumption_of_Junk_Fried_Foods | Categorical | - | 1.748726 | 0.599374 | 2 | 2 | 2 |
| Consumption_of_Sweets | Categorical | - | 1.667798 | 0.67974 | 2 | 2 | 2 |
| Consumption_of_Red_Meat | Categorical | - | 1.792303 | 0.408554 | 2 | 2 | 2 |
| Consumption_of_BBQ_Foods | Categorical | - | 1.431805 | 0.496609 | 1 | 1 | 2 |
| Consumption_of_Discritionary_Foods | Categorical | - | 1.333899 | 0.882380 | 0 | 2 | 2 |
| Existing_Health_Problems | Categorical | - | 11.74702 | 9.372253 | 7 | 14 | 14 |
| Hospitalization_History | Categorical | - | 0.220713 | 0.414844 | 0 | 0 | 0 |
| Regular_Physician_Consultation | Categorical | - | 0.398415 | 0.489710 | 0 | 0 | 1 |
| Negative_Lifestyle | Categorical | - | 0.163554 | 0.163554 | 0 | 0 | 0 |
| Skipping_Diet | Categorical | - | 1.292586 | 0.470977 | 1 | 1 | 2 |
| Food_Type | Categorical | - | 0.256367 | 0.436750 | 0 | 0 | 1 |
| WFH_for_COVID | Categorical | - | 1.024335 | 0.979967 | 0 | 1 | 2 |
| Sedentary_Lifestyle_due_to_COVID | Categorical | - | 0.331070 | 0.470731 | 0 | 0 | 1 |
| Unhealthy_Lifestyle_due_to_COVID | Categorical | - | 0.156763 | 0.363680 | 0 | 0 | 0 |
| Addiction_due_to_COVID | Categorical | - | 0.010187 | 0.100443 | 0 | 0 | 0 |
| Depressive_State | Categorical | - | 0.395020 | 0.488993 | 0 | 0 | 1 |
