# Supplementary material for: Exploring online public survey lifestyle datasets with statistical analysis, machine learning and semantic ontology
Source: Sci Rep. 2024 Oct 15;14:24190. doi: 10.1038/s41598-024-74539-6 (PMC11480510; doi:10.1038/s41598-024-74539-6)
Supplement: Supplementary file 2 — Supplementary Material 2 [file 41598_2024_74539_MOESM2_ESM.docx]

**Table S2.1**. The description of the asked questions.

| **Questions** | **Entry type** | **Choice type** | **Type** |
| --- | --- | --- | --- |
| Please Enter Your Age (in Years) | Textbox | - | Non-categorical |
| Please Enter Your Height (in inch) | Textbox | - | Non-categorical |
| Please Enter Your Weight (in Kg) | Textbox | - | Non-categorical |
| What is Your Gender. | Checkbox | Male, Female | Categorical |
| Name of the city | Textbox | - | Categorical |
| What is Your Educational Level? | Checkbox | Below 10^th^, 10th - 12^th^, Diploma, Bachelors, Masters, Above Masters | Categorical |
| Please Enter Your Economic Status | Checkbox | Poverty Line, Lower Middle Class, Middle Class, Upper Middle Class, Rich | Categorical |
| How Many Dependents Do You Have in Your Family? | Textbox | - | Non-categorical |
| Are You Employed? | Checkbox | Full-Time, Housewife, Business, Part-Time, Student, Unemployed, Retired | Categorical |
| Do You Have Medical Insurance? | Checkbox | Yes, No | Categorical |
| Do You go to Gym? | Checkbox | Yes, No | Categorical |
| How Long Do You Exercise Daily? | Checkbox | < 30 mins, 30 mins - 1 hr, 1 hr - 1.5 hrs, 1.5 hrs - 2 hrs, > 2 hrs. | Categorical |
| How Long Do You Walk Daily? | Checkbox | < 1 Km., 1 Km - 2 Km., 2 Km - 3 Km., 3 Km - 4 Km., > 4 Km | Categorical |
| Smoking Habit? (In a month) | Checkbox | Never, Occasionally, Daily | Categorical |
| The habit of Taking Snus (Khaini)? (In a month) | Checkbox | Never, Occasionally, Daily | Categorical |
| The habit of Taking Paan-Masala? (In a month) | Checkbox | Never, Occasionally, Daily | Categorical |
| The habit of Taking Alcohol? (In a month) | Checkbox | Never, Occasionally, Daily | Categorical |
| The habit of Taking Energy Drinks / Sweet Beverages (coco-cola or similar)? (In a month) | Checkbox | Never, Occasionally, Daily | Categorical |
| Are You Using Any Mobile Application for Activity Tracking? | Checkbox | Yes, No | Categorical |
| Are You Using Any Mobile Application for Diet Tracking? | Checkbox | Yes, No | Categorical |
| What Type of Social Participation Do you prefer most? | Checkbox | Offline (gathering, party), Online (social sites), Both | Categorical |
| In What Type of Social Participation Do You Spend Most of The Time in a Week? | Checkbox | Offline (gathering, party),  Online (social sites) | Categorical |
| The habit of Taking Vegetables? (In a month) | Checkbox | Never, Occasionally, Daily | Categorical |
| The habit of Taking Fruits? (In a month) | Checkbox | Never, Occasionally, Daily | Categorical |
| The habit of Taking Junk Fried Foods? (In a month) | Checkbox | Never, Occasionally, Daily | Categorical |
| The habit of Taking Sweets? (In a month) | Checkbox | Never, Occasionally, Daily | Categorical |
| The habit of Taking BBQ? (In a month) | Checkbox | Never, Occasionally, Daily | Categorical |
| The habit of Taking Cakes, Pastries, Chips, Ice-Cream, Chocolate, Jam, Butter, Energy bars, Cream? (In a month) | Checkbox | Never, Occasionally, Daily | Categorical |
| Duration of Sleep in Weekdays (Total hrs / 5) | Textbox | - | Non-categorical |
| Duration of Sleep in the Weekends (Total hrs / 2) | Textbox | - | Non-categorical |
| Are You Suffering from Any of the Below? (Diagnosed in last 1 Year or before) | Checkbox (can select more than one) | Obesity, Over-Weight, Under-Weight, Cardiovascular Diseases, Diabetes Type II, High Lipid, High BP, Depression, Sleeping Disorder, None | Categorical |
| Have You Been Hospitalized in the Last 1 Year? | Checkbox | Yes, No | Categorical |
| Do You Consult with Your Physician Regularly (every 3-6 months) As a Part of Health Check-up? | Checkbox | Yes, No | Categorical |
| Have You Observed a Negative lifestyle in You in the last 5 years? | Checkbox | Yes, No | Categorical |
| Do You Skip Your Breakfast/Lunch/Dinner Due to Work? (In a month) | Checkbox | Yes, No | Categorical |
| Which One of The Below Is Appropriate for You Currently? | Checkbox | Pregnant, Kid, Children, Disabled, Hospitalized, None | Categorical |
| Which One of The Below Is Appropriate for You? | Checkbox | Yes, No | Categorical |
| Are you working from home due to COVID-19? | Checkbox | Yes, No, Sometimes | Categorical |
| Has your life become sedentary due to COVID-19? | Checkbox | Yes, No | Categorical |
| Has your life become full of unhealthy foods due to COVID-19? | Checkbox | Yes, No | Categorical |
| Have you become more addicted to tobacco/+ alcohol due to COVID-19? | Checkbox | Yes, No | Categorical |
| Are you depressed due to the consequences of COVID-19? (e.g., confinement, no tour, no hike, jobless, no exam and poor lifestyle) | Checkbox | Yes, No | Categorical |
