# Supplementary material for: Exploring online public survey lifestyle datasets with statistical analysis, machine learning and semantic ontology
Source: Sci Rep. 2024 Oct 15;14:24190. doi: 10.1038/s41598-024-74539-6 (PMC11480510; doi:10.1038/s41598-024-74539-6)
Supplement: Supplementary file 3 — Supplementary Material 3 [file 41598_2024_74539_MOESM3_ESM.docx]

@prefix : <http://www.semanticweb.org/ayan1/ontologies/2023/0/untitled-ontology-6#> .

@prefix owl: <http://www.w3.org/2002/07/owl#> .

@prefix rdf: <http://www.w3.org/1999/02/22-rdf-syntax-ns#> .

@prefix xml: <http://www.w3.org/XML/1998/namespace> .

@prefix xsd: <http://www.w3.org/2001/XMLSchema#> .

@prefix rdfs: <http://www.w3.org/2000/01/rdf-schema#> .

@base <http://www.semanticweb.org/ayan1/ontologies/2023/0/untitled-ontology-6> .

<http://www.semanticweb.org/ayan1/ontologies/2023/0/untitled-ontology-6> rdf:type owl:Ontology .

#################################################################

# Classes

#################################################################

### http://www.semanticweb.org/ayan1/ontologies/2023/0/untitled-ontology-6#Activity

:Activity rdf:type owl:Class ;

rdfs:subClassOf :Lifestyle .

### http://www.semanticweb.org/ayan1/ontologies/2023/0/untitled-ontology-6#Addiction_due_to_COVID

:Addiction_due_to_COVID rdf:type owl:Class ;

rdfs:subClassOf :Health_Situation_During_COVID_19 .

### http://www.semanticweb.org/ayan1/ontologies/2023/0/untitled-ontology-6#Age

:Age rdf:type owl:Class ;

rdfs:subClassOf :Personal .

### http://www.semanticweb.org/ayan1/ontologies/2023/0/untitled-ontology-6#Alcohol_Consumption

:Alcohol_Consumption rdf:type owl:Class ;

rdfs:subClassOf :Habit .

### http://www.semanticweb.org/ayan1/ontologies/2023/0/untitled-ontology-6#Consumption_of_BBQ_Foods

:Consumption_of_BBQ_Foods rdf:type owl:Class ;

rdfs:subClassOf :Dietary_Habit .

### http://www.semanticweb.org/ayan1/ontologies/2023/0/untitled-ontology-6#Consumption_of_Discritionary_Foods

:Consumption_of_Discritionary_Foods rdf:type owl:Class ;

rdfs:subClassOf :Dietary_Habit .

### http://www.semanticweb.org/ayan1/ontologies/2023/0/untitled-ontology-6#Consumption_of_Fruits

:Consumption_of_Fruits rdf:type owl:Class ;

rdfs:subClassOf :Dietary_Habit .

### http://www.semanticweb.org/ayan1/ontologies/2023/0/untitled-ontology-6#Consumption_of_Junk_Fried_Foods

:Consumption_of_Junk_Fried_Foods rdf:type owl:Class ;

rdfs:subClassOf :Dietary_Habit .

### http://www.semanticweb.org/ayan1/ontologies/2023/0/untitled-ontology-6#Consumption_of_Red_Meat

:Consumption_of_Red_Meat rdf:type owl:Class ;

rdfs:subClassOf :Dietary_Habit .

### http://www.semanticweb.org/ayan1/ontologies/2023/0/untitled-ontology-6#Consumption_of_Sweets

:Consumption_of_Sweets rdf:type owl:Class ;

rdfs:subClassOf :Dietary_Habit .

### http://www.semanticweb.org/ayan1/ontologies/2023/0/untitled-ontology-6#Consumption_of_Vegetables

:Consumption_of_Vegetables rdf:type owl:Class ;

rdfs:subClassOf :Dietary_Habit .

### http://www.semanticweb.org/ayan1/ontologies/2023/0/untitled-ontology-6#Diet

:Diet rdf:type owl:Class ;

rdfs:subClassOf :Lifestyle .

### http://www.semanticweb.org/ayan1/ontologies/2023/0/untitled-ontology-6#Dietary_Habit

:Dietary_Habit rdf:type owl:Class ;

rdfs:subClassOf :Diet .

### http://www.semanticweb.org/ayan1/ontologies/2023/0/untitled-ontology-6#Duration_of_daily_exercise

:Duration_of_daily_exercise rdf:type owl:Class ;

rdfs:subClassOf :Physical_Activity .

### http://www.semanticweb.org/ayan1/ontologies/2023/0/untitled-ontology-6#Duration_of_daily_walking

:Duration_of_daily_walking rdf:type owl:Class ;

rdfs:subClassOf :Physical_Activity .

### http://www.semanticweb.org/ayan1/ontologies/2023/0/untitled-ontology-6#Economic_Status

:Economic_Status rdf:type owl:Class ;

rdfs:subClassOf :Personal .

### http://www.semanticweb.org/ayan1/ontologies/2023/0/untitled-ontology-6#Educational_Level

:Educational_Level rdf:type owl:Class ;

rdfs:subClassOf :Personal .

### http://www.semanticweb.org/ayan1/ontologies/2023/0/untitled-ontology-6#Existing_Health_Problems

:Existing_Health_Problems rdf:type owl:Class ;

rdfs:subClassOf :Medical_History .

### http://www.semanticweb.org/ayan1/ontologies/2023/0/untitled-ontology-6#Food_Type

:Food_Type rdf:type owl:Class ;

rdfs:subClassOf :Dietary_Habit .

### http://www.semanticweb.org/ayan1/ontologies/2023/0/untitled-ontology-6#Gender

:Gender rdf:type owl:Class ;

rdfs:subClassOf :Personal .

### http://www.semanticweb.org/ayan1/ontologies/2023/0/untitled-ontology-6#Habit

:Habit rdf:type owl:Class ;

rdfs:subClassOf :Lifestyle .

### http://www.semanticweb.org/ayan1/ontologies/2023/0/untitled-ontology-6#Habit_of_Alcohol

:Habit_of_Alcohol rdf:type owl:Class ;

rdfs:subClassOf :Tobacco_Consumption .

### http://www.semanticweb.org/ayan1/ontologies/2023/0/untitled-ontology-6#Habit_of_Paan_Masala

:Habit_of_Paan_Masala rdf:type owl:Class ;

rdfs:subClassOf :Habit .

### http://www.semanticweb.org/ayan1/ontologies/2023/0/untitled-ontology-6#Habit_of_Snus

:Habit_of_Snus rdf:type owl:Class ;

rdfs:subClassOf :Tobacco_Consumption .

### http://www.semanticweb.org/ayan1/ontologies/2023/0/untitled-ontology-6#Health_Situation_During_COVID_19

:Health_Situation_During_COVID_19 rdf:type owl:Class ;

rdfs:subClassOf :Questionnaire_Type .

### http://www.semanticweb.org/ayan1/ontologies/2023/0/untitled-ontology-6#Height

:Height rdf:type owl:Class ;

rdfs:subClassOf :Personal .

### http://www.semanticweb.org/ayan1/ontologies/2023/0/untitled-ontology-6#Hospitalization_History

:Hospitalization_History rdf:type owl:Class ;

rdfs:subClassOf :Medical_History .

### http://www.semanticweb.org/ayan1/ontologies/2023/0/untitled-ontology-6#Lifestyle

:Lifestyle rdf:type owl:Class ;

rdfs:subClassOf :Questionnaire_Type .

### http://www.semanticweb.org/ayan1/ontologies/2023/0/untitled-ontology-6#Medical_History

:Medical_History rdf:type owl:Class ;

rdfs:subClassOf :Questionnaire_Type .

### http://www.semanticweb.org/ayan1/ontologies/2023/0/untitled-ontology-6#Mobile_Application_for_Activity_Tracking

:Mobile_Application_for_Activity_Tracking rdf:type owl:Class ;

rdfs:subClassOf :Personal .

### http://www.semanticweb.org/ayan1/ontologies/2023/0/untitled-ontology-6#Mobile_Application_for_Diet_Tracking

:Mobile_Application_for_Diet_Tracking rdf:type owl:Class ;

rdfs:subClassOf :Personal .

### http://www.semanticweb.org/ayan1/ontologies/2023/0/untitled-ontology-6#Negative_Lifestyle

:Negative_Lifestyle rdf:type owl:Class ;

rdfs:subClassOf :Lifestyle .

### http://www.semanticweb.org/ayan1/ontologies/2023/0/untitled-ontology-6#Personal

:Personal rdf:type owl:Class ;

rdfs:subClassOf :Questionnaire_Type .

### http://www.semanticweb.org/ayan1/ontologies/2023/0/untitled-ontology-6#Physical_Activity

:Physical_Activity rdf:type owl:Class ;

rdfs:subClassOf :Activity .

### http://www.semanticweb.org/ayan1/ontologies/2023/0/untitled-ontology-6#Questionnaire_Type

:Questionnaire_Type rdf:type owl:Class .

### http://www.semanticweb.org/ayan1/ontologies/2023/0/untitled-ontology-6#Regular_Physician_Consultation

:Regular_Physician_Consultation rdf:type owl:Class ;

rdfs:subClassOf :Medical_History .

### http://www.semanticweb.org/ayan1/ontologies/2023/0/untitled-ontology-6#Sedentary_Lifestyle_due_to_COVID

:Sedentary_Lifestyle_due_to_COVID rdf:type owl:Class ;

rdfs:subClassOf :Health_Situation_During_COVID_19 .

### http://www.semanticweb.org/ayan1/ontologies/2023/0/untitled-ontology-6#Skipping_Diet

:Skipping_Diet rdf:type owl:Class ;

rdfs:subClassOf :Diet .

### http://www.semanticweb.org/ayan1/ontologies/2023/0/untitled-ontology-6#Sleeping_Habit

:Sleeping_Habit rdf:type owl:Class ;

rdfs:subClassOf :Activity .

### http://www.semanticweb.org/ayan1/ontologies/2023/0/untitled-ontology-6#Smoking_Habit

:Smoking_Habit rdf:type owl:Class ;

rdfs:subClassOf :Tobacco_Consumption .

### http://www.semanticweb.org/ayan1/ontologies/2023/0/untitled-ontology-6#Social

:Social rdf:type owl:Class ;

rdfs:subClassOf :Questionnaire_Type .

### http://www.semanticweb.org/ayan1/ontologies/2023/0/untitled-ontology-6#Social_Participation_Duration

:Social_Participation_Duration rdf:type owl:Class ;

rdfs:subClassOf :Social .

### http://www.semanticweb.org/ayan1/ontologies/2023/0/untitled-ontology-6#Social_Participation_Type

:Social_Participation_Type rdf:type owl:Class ;

rdfs:subClassOf :Social .

### http://www.semanticweb.org/ayan1/ontologies/2023/0/untitled-ontology-6#State_of_Depression

:State_of_Depression rdf:type owl:Class ;

rdfs:subClassOf :Questionnaire_Type .

### http://www.semanticweb.org/ayan1/ontologies/2023/0/untitled-ontology-6#Sweet_Beverages_Consumption

:Sweet_Beverages_Consumption rdf:type owl:Class ;

rdfs:subClassOf :Dietary_Habit .

### http://www.semanticweb.org/ayan1/ontologies/2023/0/untitled-ontology-6#Tobacco_Consumption

:Tobacco_Consumption rdf:type owl:Class ;

rdfs:subClassOf :Habit .

### http://www.semanticweb.org/ayan1/ontologies/2023/0/untitled-ontology-6#Unhealthy_Lifestyle_due_to_COVID

:Unhealthy_Lifestyle_due_to_COVID rdf:type owl:Class ;

rdfs:subClassOf :Health_Situation_During_COVID_19 .

### http://www.semanticweb.org/ayan1/ontologies/2023/0/untitled-ontology-6#Visit_Gym

:Visit_Gym rdf:type owl:Class ;

rdfs:subClassOf :Physical_Activity .

### http://www.semanticweb.org/ayan1/ontologies/2023/0/untitled-ontology-6#WFH_for_COVID

:WFH_for_COVID rdf:type owl:Class ;

rdfs:subClassOf :Health_Situation_During_COVID_19 .

### http://www.semanticweb.org/ayan1/ontologies/2023/0/untitled-ontology-6#Week_Days_sleep_time_avg

:Week_Days_sleep_time_avg rdf:type owl:Class ;

rdfs:subClassOf :Sleeping_Habit .

### http://www.semanticweb.org/ayan1/ontologies/2023/0/untitled-ontology-6#Weekends_sleep_time_avg

:Weekends_sleep_time_avg rdf:type owl:Class ;

rdfs:subClassOf :Sleeping_Habit .

### http://www.semanticweb.org/ayan1/ontologies/2023/0/untitled-ontology-6#Weight

:Weight rdf:type owl:Class ;

rdfs:subClassOf :Personal .

### Generated by the OWL API (version 4.5.9.2019-02-01T07:24:44Z) https://github.com/owlcs/owlapi
